# Exploring the distribution and co-occurrence of *rpf*-like genes and nitrogen-cycling genes in water reservoir sediments

**DOI:** 10.3389/fmicb.2024.1433046

**Published:** 2024-07-22

**Authors:** Aiqin Hou, Huayi Fu, Leilei Liu, Xiaomei Su, Shusheng Zhang, Jiahou Lai, Faqian Sun

**Affiliations:** ^1^College of Geography and Environmental Science, Zhejiang Normal University, Jinhua, China; ^2^The Management Center of Wuyanling National Natural Reserve in Zhejiang, Wenzhou, China

**Keywords:** reservoir sediments, ***rpf***-like genes, nitrogen-cycling genes, microbial community, co-occurrence network

## Abstract

Water reservoir sediments represent a distinct habitat that harbors diverse microbial resources crucial for nitrogen cycling processes. The discovery of resuscitation promoting factor (Rpf) has been recognized as a crucial development in understanding the potential of microbial populations. However, our understanding of the relationship between microorganisms containing *rpf*-like genes and nitrogen-cycling functional populations remains limited. The present study explored the distribution patterns of *rpf*-like genes and nitrogen-cycling genes in various water reservoir sediments, along with their correlation with environmental factors. Additionally, the co-occurrence of *rpf*-like genes with genes associated with the nitrogen cycle and viable but non-culturable (VBNC) formation was investigated. The findings indicated the ubiquitous occurrence of Rpf-like domains and their related genes in the examined reservoir sediments. Notably, *rpf*-like genes were predominantly associated with *Bradyrhizobium*, *Nitrospira*, and *Anaeromyxobacter*, with pH emerging as the primary influencing factor for their distribution. Genera such as *Nitrospira*, *Bradyrhizobium*, *Anaeromyxobacter*, and *Dechloromonas* harbor the majority of nitrogen-cycling functional genes, particularly denitrification genes. The distribution of nitrogen-cycling microbial communities in the reservoir sediments was mainly influenced by pH and NH_4_^+^. Notably, correlation network analysis revealed close connections between microorganisms containing *rpf*-like genes and nitrogen-cycling functional populations, as well as VBNC bacteria. These findings offer new insights into the prevalence of *rpf*-like genes in the water reservoir sediments and their correlation with nitrogen-cycling microbial communities, enhancing our understanding of the significant potential of microbial nitrogen cycling.

## 1 Introduction

Reservoirs, formed by the construction of dams for purposes such as hydroelectricity production, flood control, and recreation activities, offer a distinctive ecological habitat (Zhang et al., 2015; Yang et al., 2021). The significance of reservoirs has grown in meeting the increasing energy and water demands. The importance of maintaining favorable chemical and biological water quality in reservoirs cannot be overstated, as it not only helps in reducing treatment costs but also ensures the supply of safe drinking water. Acting as both sinks and sources in nutrient cycles within water reservoirs, sediments serve as vital components of element migration and transformation (Zhang et al., 2015; Li et al., 2019). Microorganisms in water reservoir sediments, which exhibit high diversity and variability, are crucial for pollutant degradation and nutrient biogeochemical cycling, contributing significantly to the overall health and functioning of reservoir ecosystems (Gao et al., 2021; Wu et al., 2021).

Considerable attention has been directed towards examining the distribution and dynamics of functional microbial populations in reservoir sediments, particularly those involved in nitrogen cycling (Yang et al., 2021; Zhang et al., 2021; Lv et al., 2022; Shi et al., 2022). Microbe-driven nitrogen cycling encompasses various distinct processes, such as nitrogen fixation, nitrification, denitrification, anaerobic ammonium oxidation (anammox), dissimilatory/assimilatory nitrate reduction to ammonium (DNRA/ANRA), and ammonia assimilation/ammonification (Kuypers et al., 2018; Wang et al., 2022a; Nie et al., 2023; Zhang et al., 2023c). Previous research has detected microorganisms involved in these processes in reservoir ecosystems using molecular methods. However, most of these studies have focused on specific nitrogen cycling processes or functional populations by targeting limited genes through high-throughput amplicon sequencing and/or quantitative real-time PCR methods (Wang et al., 2021; Sun et al., 2023; Yuan et al., 2023). Comprehensive investigations of various nitrogen-cycling functional genes in water reservoir sediments using metagenomic approaches are still scarce. Thus, it is essential to conduct a comprehensive investigation into the functional microbial communities that participate in nitrogen cycles within reservoir sediments.

Additionally, water reservoir sediments serve as a unique habitat harboring diverse microbial resources. However, the identification of functional strains within these sediments remains limited. It should be noted that the majority of microorganisms on Earth are inaccessible and cannot be cultivated in pure cultures, with less than 1% being successfully cultured (Lin et al., 2023; Zhou et al., 2023). A significant challenge in obtaining functional strains arises from the presence of microorganisms in the viable but non-culturable (VBNC) state, which are incapable of forming colonies on culture plates (Su et al., 2018; Han et al., 2023). The VBNC state, similar to other non-growing states like persistence and dormancy, is a common occurrence for long-term survival under stressful conditions (Xie et al., 2021; Lin et al., 2023). The conditions that trigger the VBNC state, such as extreme temperatures, osmotic pressure, pH fluctuations, starvation, and high concentrations of pollutants, are commonly found in natural environments (Han et al., 2023; Yang et al., 2024). To date, more than 100 microbial species have been documented to enter the VBNC state (Su et al., 2023). VBNC microorganisms have been recognized in diverse environments, and the resuscitation of these microorganisms plays a vital role in environmental bioremediation and biogeochemical cycles (Lennon and Jones, 2011; Shi et al., 2024a).

The discovery of resuscitation promoting factor (Rpf) from *Micrococcus luteus* has been a significant breakthrough in the recovery of VBNC microorganisms, due to its potent anti-dormancy effects at picomolar concentrations (Su et al., 2023; Zhou et al., 2023). Studies have demonstrated its ability to resuscitate and enhance the activity of functional populations engaged in the degradation of diverse xenobiotics, including phenol (Su et al., 2018), polychlorinated biphenyls (PCBs) (Su et al., 2019c), and anthraquinone dyes (Cai et al., 2021). Notably, a strain *Pseudomonas* sp. SSPR1, resuscitated through Rpf supplementation, exhibited excellent performance in simultaneous nitrification and denitrification (Su et al., 2019b). The Rpf-responsive bacterial communities are distributed across phyla *Actinobacteria*, *Proteobacteria*, *Bacteroidetes*, and *Firmicutes*, encompassing numerous functional populations associated with biogeochemical cycles (Su et al., 2019a, 2023; Zhou et al., 2023). Furthermore, *rpf*-like genes have been identified in various genera, such as *Rhodococcus*, *Mycobacterium*, *Streptomyces*, *Achromobacter*, *Nocardia*, *Corynebacterium*, and *Saccharopolyspora*, exhibiting similar muralytic activity (Mukamolova et al., 2006; Kana et al., 2008; Hoang et al., 2021; Shi et al., 2024b). These genes are widely distributed across various environments and are closely linked to the transition between the VBNC and active states in microbial communities (Lennon and Jones, 2011; Shi et al., 2024b). Although previous studies have explored the use of Rpf homologues in vaccine development and bioremediation enhancements (Kana et al., 2008; Su et al., 2018; Zhou et al., 2023), their presence in natural environments and their potential correlation with nitrogen-cycling genes have yet to be investigated. Therefore, exploring the distribution and co-occurrence of *rpf*-like genes with the nitrogen cycle and VBNC formation in water reservoir sediments is essential.

In this study, three water reservoirs located in Wuyanling, a national-level nature reserve in China nearest to the East China Sea, were chosen for investigating sediment-associated functional microbial communities through metagenomic sequencing. The study aimed to: (1) demonstrate the structure and composition of sediment microbial communities and their correlation with environmental factors in the three water reservoirs; (2) analyze the distribution patterns of *rpf*-like genes and nitrogen-cycling genes and their association with environmental factors in diverse water reservoir sediments; (3) explore the co-occurrence of *rpf*-like genes with genes involved in the nitrogen cycle and VBNC formation. The present study is the first to report on the presence of *rpf*-like genes in water reservoir sediments and their association with functional bacterial populations participating in nitrogen cycles. These findings shed light on the crucial role of sediment microbes in nitrogen cycles within water reservoirs and highlight the potential of Rpf-like proteins in stimulating functional populations.

## 2 Materials and methods

### 2.1 Study site and sampling

During the optimal season for nitrogen cycling processes, sediment sampling was conducted in July 2022 at three water reservoirs, namely Sanchaxi (SCX), Zhangnenzi (ZNZ), and Wenyang (WY), located in the Wuyanling National Nature Reserve in the Zhejiang Province, China. These reservoirs are crucial for hydroelectricity production, with SCX being the largest among them. A total of five sampling sites (SCX1–SCX5) were designated for SCX, while three sampling sites were designed for both ZNZ (ZNZ1–ZNZ3) and WY (WY1–WY3). The sampling points within each reservoir were sequentially numbered from upstream to downstream. Detailed information regarding the sampling sites can be found in Supplementary Figure S1. At each sampling site, surface sediments were collected from the 0–30 cm depth using a Petersen stainless steel grab sampler. To ensure sample representativeness, a composite surface sediment sample was created by combining three sediment samples collected in close proximity to the sampling site (Zhang et al., 2015; Gao et al., 2021). Additionally, corresponding water samples were collected using a vertical water sampler. The sediment samples were sealed in sample bags, and the water samples were stored in polyethylene bottles. All samples were then placed in ice boxes for transportation to the laboratory. Subsequently, the water samples underwent immediate analysis of their physicochemical characteristics, while the sediment samples were stored separately in sampling bags (at 4°C) and polypropylene tubes (at −80°C) for further analysis of physicochemical characteristics and microbial communities, respectively.

### 2.2 Physicochemical characteristics analysis

The sediment pH was measured using a PHSJ-4F pH meter (Leici, China) with a sediment suspension ratio of 1:2.5 (sediment to deionized water). Prior to measurement, the deionized water was purged with nitrogen gas for 15 min. The moisture content (MC) of sediments was assessed by oven-drying fresh sediment at 105°C until a constant weight was achieved (Wang et al., 2021). To extract exchangeable dissolved inorganic nitrogen, including ammonium (NH_4_^+^), nitrite (NO_2_^−^), and nitrate (NO_3_^−^), from fresh sediments, a 2 M KCl solution pre-purged with high-purity nitrogen gas was utilized at a ratio of 1:5 (sediment to KCl solution, w/v). The resulting solution was filtered through a 0.45 μm membrane filter and then measured using a TU-1810 UV/VIS spectrophotometer, following the standard method (Li et al., 2019). The total organic matter (TOM) content was estimated by measuring the weight loss after combustion at 550°C using the LOI550 (loss on ignition at 550°C) method (Wang et al., 2021). Additionally, water quality parameters including pH, NH_4_^+^, NO_2_^−^, NO_3_^−^, total phosphorus, total organic carbon, and total nitrogen were determined using standard methods (Rice et al., 2012). All the physicochemical characteristics were measured in triplicate. The summaries of the physicochemical characteristics of both the sediment and water samples are presented in Supplementary Tables S1, S2, respectively.

### 2.3 Metagenomic sequencing

The total metagenomic DNA of the sediment samples was extracted using FastDNA Spin Kit for Soil (MP Biomedicals, United States). The concentration of the extracted DNA was determined using the TBS-380, while the purity was assessed using the NanoDrop2000. Equal masses of DNA from each of the three replicate samples were consolidated to reduce potential variability. The DNA extract was then fragmented to an average size of approximately 400 bp using the Covaris M220 to facilitate the construction of paired-end libraries. For library construction, the NEXTflex rapid DNA-seq kit (Bioo Scientific, United States) was utilized, with adapters that included the full set of sequencing primer hybridization sites ligated to the blunt ends of the DNA fragments. Paired-end sequencing was conducted on the Illumina Novaseq 6,000 (Illumina Inc., San Diego, CA, United States) at Majorbio Bio-Pharm Technology Co., Ltd. (Shanghai, China). The raw sequence data have been deposited in the NCBI Sequence Read Archive under the project accession number PRJNA1061319.

### 2.4 Metagenome assembly and annotation

To ensure the reliability of the metagenomic data, adaptors and low-quality reads were trimmed. The remaining high-quality reads were then assembled using MEGAHIT, with contigs ≥300 bp chosen as the ultimate assembly. Gene annotation and prediction were performed on the contigs. Gene abundance quantification was conducted through the reads per kilobase per million mapped reads (RPKM) method. For each contig, open reading frames (ORFs) were predicted using MetaGene (Noguchi et al., 2006), and those with a length of ≥100 bp were extracted for translation into amino acid sequences using the NCBI translation table. Subsequently, a non-redundant gene catalog was built using CD-HIT, applying a 95% sequence identity and 90% coverage threshold. The high-quality reads were then aligned to this non-redundant gene catalog using SOAPaligner for determining gene abundance with 95% identity. For taxonomic annotations, representative sequences from the non-redundant gene catalog were aligned to the NR database using Diamond (Buchfink et al., 2015). The alpha diversity of the microbial community was assessed by calculating the number of reads at the genus level. The Conserved Domain Database (CDD) was utilized to annotate the structural domains of non-redundant genes (Jia et al., 2022; Shi et al., 2024b). The functions of non-redundant genes were annotated against the Kyoto Encyclopedia of Genes and Genomes (KEGG) database using Diamond (Buchfink et al., 2015).

### 2.5 Identification of rpf-like genes and genes associated with the nitrogen cycle and VBNC formation

The presence of *rpf*-like genes in the water reservoir sediments was investigated by analyzing the detected Rpf-like domains (Supplementary Table S3), including LT_TF-like, chitinase_GH19, lyz_endolysin_autolysin, chitosanase_GH46, pesticin_lyz-like, N-acetylmuramidase_GH108, lambda_lys-like, LT-like, Slt35-like, LT_IagB-like, Slt70-like, MLTF-like, LT_VirB1-like, LT_MltC_MltE, LT_Slt70-like, MltD-like, and Lyz-like.^1^ Moreover, an investigation was conducted on the distribution of nitrogen-cycling genes (Supplementary Table S4) encompassing nitrogen fixation (*nifH*, *nifD*, *nifK*), nitrification (*amoA/pmoA*, *amoB/pmoB*, *amoC/pmoC*, *hao*, *nxrB*), denitrification (*narG*, *nirS*, *nirK*, *nosZ*, *norB*), DNRA (*nrfA*, *nirB*, *nirD*, *nrfH*), ANRA (*nasA*, *nirA*, *narB*), anammox (*hzsA_1*/*hzsA_2*, *hdh/hzo*, *hzsB*, *hzsC*), and ammonification (*ureC*, *gdhA*) in the water reservoir sediments (Kuypers et al., 2018; Zhang et al., 2022; Wang et al., 2022a). Additionally, the genes involved in VBNC formation (Supplementary Table S5) were also examined in the water reservoir sediments to explore their potential co-occurrence with *rpf*-like genes and nitrogen-cycling functional genes.

### 2.6 Statistical analysis

The physicochemical properties data obtained from the experiment in triplicate were presented as mean values and standard deviations. Principal coordinates analysis (PCoA) with Bray–Curtis dissimilarity was utilized to assess the dissimilarity of sediment microbial communities from three water reservoirs (Zhou et al., 2023). The significance of these differences was evaluated using the analysis of similarities (ANOSIM) test. To compare the microbial community variations among the three water reservoir sediments, the Kruskal–Wallis H test was performed, followed by the Tukey–Kramer test at a 95% confidence level. Redundancy analysis (RDA) was employed to examine the association between environmental variables and functional genes, as well as their associated microbial communities. The environmental variables considered in the RDA included pH, NH_4_^+^, NO_2_^−^, NO_3_^−^, and TOM, which were selected to avoid collinearity and assessed using variance inflation factors. The significance of the RDA analysis was determined using Permutest (Shi et al., 2022). The relationships between functional genes and environmental factors, as well as dominant taxa and environmental factors, were evaluated using Spearman’s correlation coefficient (Wang et al., 2022a). Mantel test was employed to determine the correlation between different microbial communities and environmental factors. Additionally, to mitigate collinearity effects among variables, only functional genes or genera present in more than half (>50%) of all 11 sediment samples were included for network construction (Liu et al., 2023). Co-occurrence networks were generated using Spearman rank correlations, setting a threshold of |*r*| ≥ 0.7 and *p* < 0.01 to investigate the co-occurrence relationships among *rpf*-like genes, and genes related to nitrogen cycle and VBNC formation, as well as among the gene-associated genera. Spearman correlation coefficients were computed using the R package “psych,” and the resulting network diagrams were visualized using the Gephi 0.9.2 platform (Liu et al., 2023).

## 3 Results and discussion

### 3.1 Microbial diversity and community composition

The physiochemical properties (Supplementary Table S1) of the sediment samples exhibited notable spatial heterogeneity at a statistically significant level (*p* < 0.05). SCX sediments were marginally alkaline, while WY sediments were slightly acidic. ZNZ sediments had varying pH levels (6.80 to 8.51), with ZNZ1 upstream being alkaline, potentially due to proximity to a village and impact of domestic wastewater. WY sediments had the highest MC values (58.26–71.39%), followed by SCX (54.45–63.94%) and ZNZ (33.54–53.93%). The trend in ammonia concentrations was WY > SCX > ZNZ, with a decrease from upstream to downstream. Nitrite concentrations followed a pattern of ZNZ > SCX > WY. There were no significant differences in NO_3_^−^ and TOM concentrations among the reservoir sediments. High-quality metagenomic sequencing data was obtained for sediment samples from the three reservoirs (Supplementary Table S6), with alpha diversity indices of microbial communities detailed in Supplementary Table S7. The Shannon diversity of sediment samples showed a significant positive correlation with NH_4_^+^ (*r* = 0.76, *p* < 0.05). Previous studies have indicated that an appropriate range of ammonium nitrogen concentration can supply necessary nutrients for microbial growth, facilitating microbial reproduction in the absence of other nitrogen sources (Kuypers et al., 2018).

**Figure 1:**
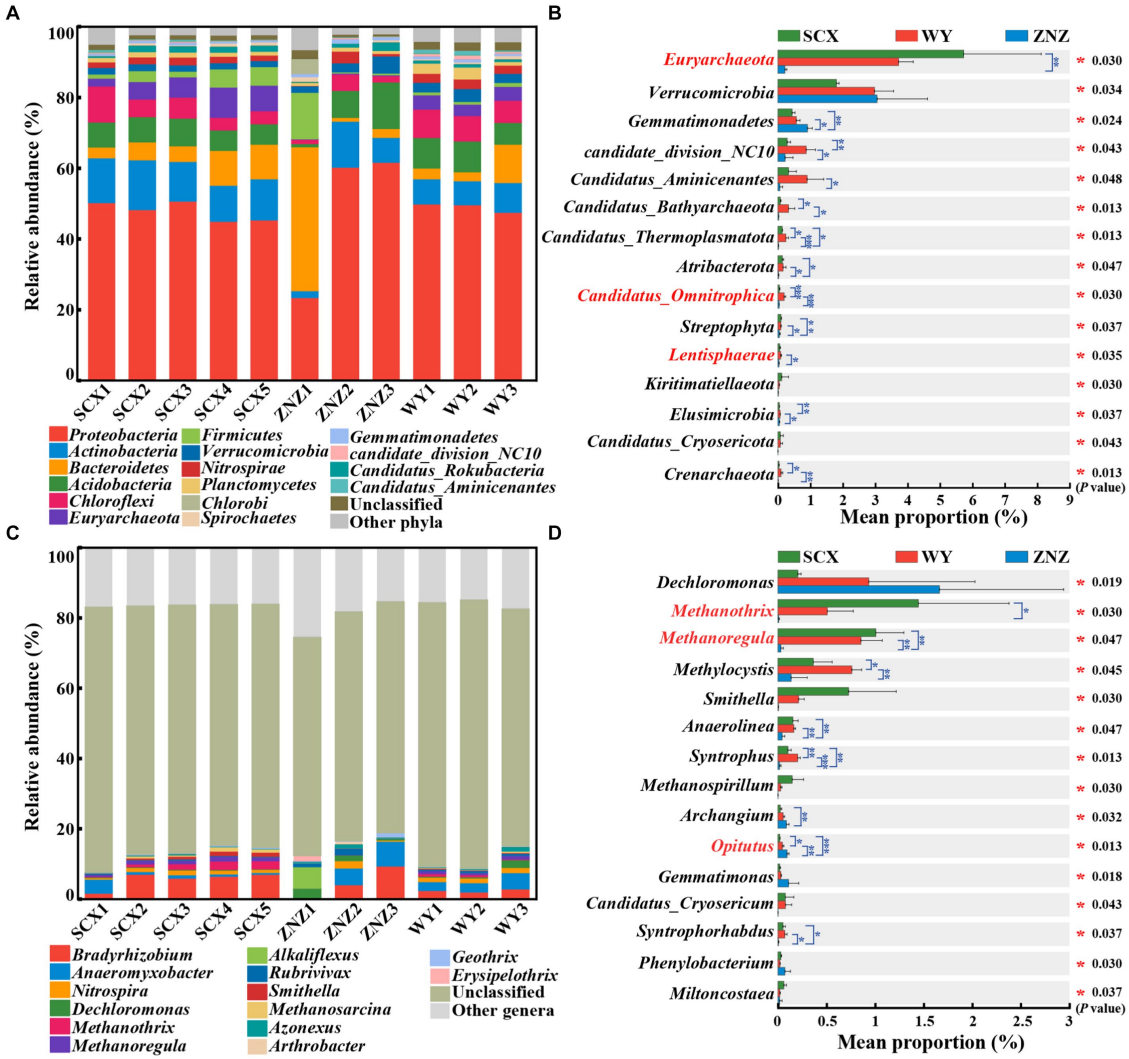
Microbial community composition and abundance in sediments from three reservoirs. **(A)** Relative abundance of microbial community at the phylum level. **(B)** Comparative analysis of the relative abundance of the top 15 most abundant microbial communities at the phylum level. **(C)** Relative abundance of microbial community at the genus level. **(D)** Comparative analysis of the relative abundance of the top 15 most abundant microbial communities at the genus level. The “Unclassified” column indicates the combined relative sequence abundances of taxonomically unclassified sequences. The “Other phyla”/"Other genera” column indicates combined relative sequence abundances of all rare phyla/genera, defined as relative sequence abundances of <1% for all samples. Significant differences are denoted by asterisks: *p < 0.05, **p < 0.01, and ***p < 0.001.

PCoA revealed significant differences in the microbial community compositions within sediment samples from the three reservoirs, as evidenced by ANOSIM test (*r* = 0.38, *p* = 0.014) (Supplementary Figure S2). Microbial communities at phylum and genus levels, with notable variations across the reservoirs, are illustrated in Figure 1. *Proteobacteria* (45.01–50.69%) was the predominant phylum in all sediment samples except ZNZ1, where *Bacteroidetes* (40.63%) dominated (Figure 1A). The prevalence of *Proteobacteria* in reservoir ecosystems is extensively documented (Li et al., 2019; Wu et al., 2021). Furthermore, significant differences (*p* < 0.05) in the relative abundance of 38 phyla were observed across the reservoirs (Figure 1B). In addition, pairwise comparisons indicated notable variations in *Euryarchaeota* between SCX and ZNZ (*p* < 0.01). *Candidatus Omnitrophica* exhibited significant differences in relative abundance between WY and SCX (*p* < 0.001), as well as between WY and ZNZ (*p* < 0.001). *Lentisphaerae* also demonstrated significant differences between WY and ZNZ (*p* < 0.05) (Figure 1B). Notably, *Euryarchaeota*, *Candidatus Omnitrophica*, and *Lentisphaerae* are known to play roles in geomicrobiological nitrogen cycling (Speth et al., 2016).

At the genus level, *Bradyrhizobium* (1.70–9.44%) and *Anaeromyxobacter* (0.52–7.03%) were the predominant genera in the three reservoirs, except for ZNZ1 (Figure 1C). These genera are known for their involvement in nitrogen fixation and the DNRA process in the nitrogen cycle (Nelson et al., 2016; Sun et al., 2023). In ZNZ1, the most predominant genus was the methane-producing bacterium *Alkaliflexus* (6.12%), favored by the alkaline conditions of ZNZ1, providing an optimal environment for the growth of *Alkaliflexus* (Wang et al., 2022b). Furthermore, *Dechloromonas* (2.95%), known as an organohalide-respiring bacterium, was the second most abundant genus in ZNZ1, associated with the degradation of aromatic compounds (Yuan et al., 2023). Additionally, based on multiple comparisons, the relative abundance of 261 genera, including *Dechloromonas*, exhibited statistically significant differences among the three reservoirs (*p* < 0.05) (Figure 1D). Pairwise comparisons revealed significant differences in *Methanothrix* between SCX and ZNZ (*p* < 0.05), and in *Methanoregula* between ZNZ and SCX (*p* < 0.01), as well as between ZNZ and WY (*p* < 0.01). Both *Methanothrix* and *Methanoregula*, members of the class *Methanomicrobia*, are known to play a crucial role in methane production (Mand and Metcalf, 2019; Wu et al., 2021). Significant variations in the relative abundance of the genus *Opitutus*, involved in the DNRA process (Nelson et al., 2016), were observed between all pairs of reservoirs (Figure 1D). It is noteworthy that a substantial proportion of the genera in the three reservoirs were unclassified (Figure 1C), indicating limited accessibility to most of them. Restoring the activity and culturability of VBNC microorganisms in the reservoirs is essential to bridge the significant gaps between functional strains obtained and those existing in the environments (Lennon and Jones, 2011; Zhou et al., 2023).

### 3.2 Distribution of *rpf*-like genes and their correlation with environmental factors

**Figure 2:**
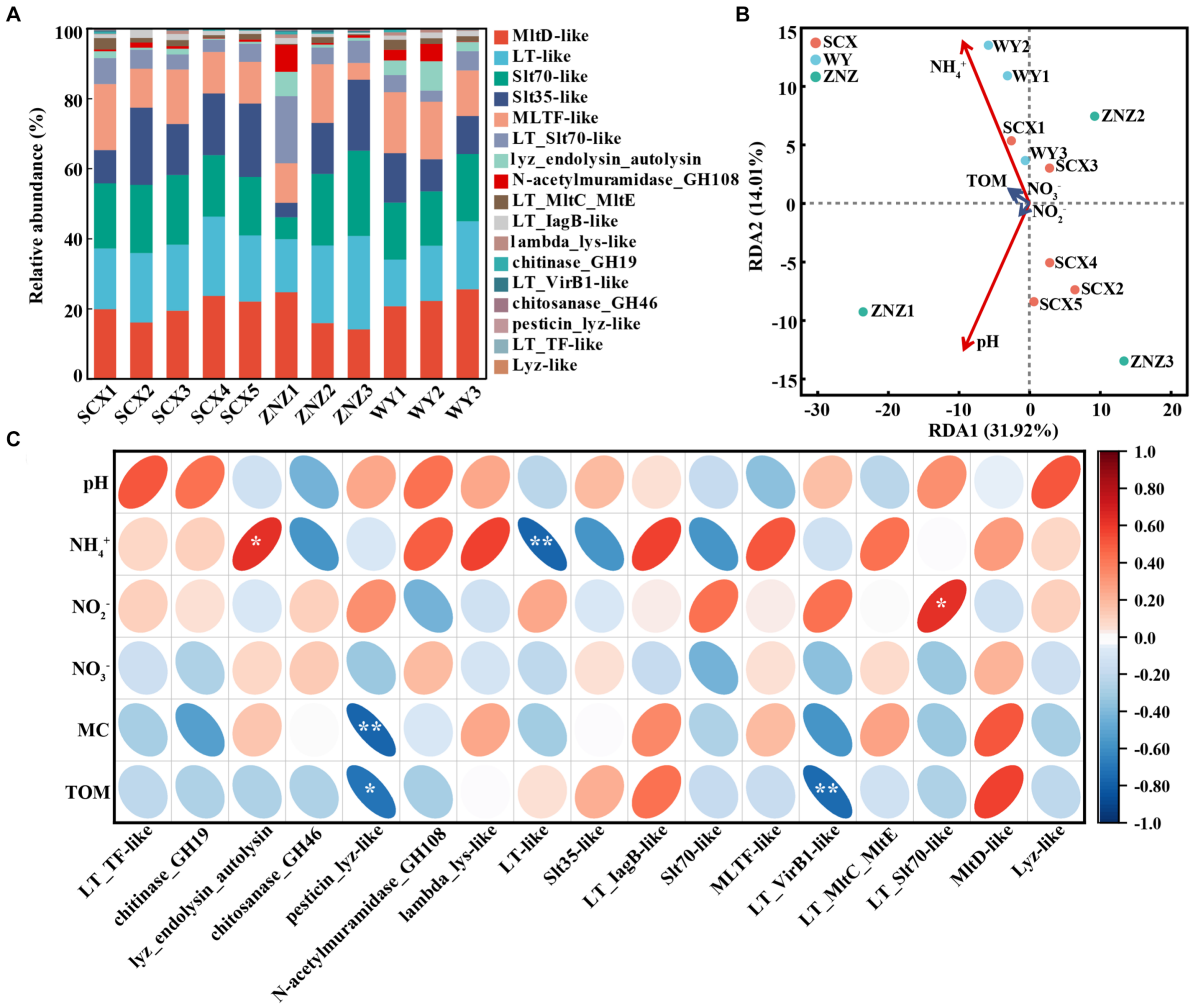
Distribution of Rpf-like domains and associated genes in sediment samples, and their correlation with environmental factors. **(A)** Percentage of abundance for Rpf-like domains and associated genes at each sampling site. **(B)** Redundancy analysis (RDA) illustrating the relationship between *rpf*-like genes and physiochemical parameters of sediments. The variables significantly influencing *rpf*-like genes, as selected by the forward selection procedure, are highlighted in red on the ordinations. **(C)** Spearman correlation analysis between *rpf*-like genes and environmental factors. The red and blue ellipses indicate positive and negative correlations, respectively. MC, moisture content; TOM, total organic matter. Significant correlations are denoted by asterisks: *p < 0.05 and **p < 0.01.

The presence of Rpf-like domains and associated genes in sediment samples from the three reservoirs is depicted in Figure 2A and detailed in Supplementary Table S3. Specifically, SCX, ZNZ, and WY contained 577, 1,246, and 327 genes respectively, spanning across 14, 17, and 14 Rpf-like domains. The samples SCX1 and ZN1, located closest to the upstream (near the village) in SCX and ZN, exhibited the greatest occurrence of *rpf*-like genes, while sample WY3 in the WY reservoir displayed the greatest occurrence of *rpf*-like genes. Notably, six Rpf-like domains, including MltD-like, Slt70-like, LT-like, MLTF-like, Slt35-like, and LT_Slt70-like, were particularly prevalent across the three reservoirs, collectively representing 87.58% of the total *rpf*-like genes. Moreover, a systematic assessment was conducted to investigate the relationship between *rpf*-like genes and environmental variables. RDA analysis showed that 45.93% of the total variation in *rpf*-like genes could be attributed to five environmental factors, including pH, NH_4_^+^, NO_2_^−^, NO_3_^−^ and TOM. Importantly, pH and NH_4_^+^ were identified as exerting significant influences (*p* < 0.05) on the distribution of *rpf*-like genes in the sediment samples (Figure 2B). Specifically, the relative abundance of lyz_endolysin_autolysin exhibited a significant positive correlation (*r* = 0.63, *p* < 0.05) with NH_4_^+^ concentration, whereas the relative abundance of LT-like showed a significant negative correlation (*r* = −0.76, *p* < 0.01) with NH_4_^+^ (Figure 2C). Additionally, pesticin_lyz-like and LT_VirB1-like were significantly associated with TOM, while LT_Slt70-like displayed a significant positive correlation (*r* = 0.63, *p* < 0.05) with NO_2_^−^ concentration. These findings suggested the significant influence of NH_4_^+^ concentration on shaping the distribution of *rpf*-like genes in sediment samples from water reservoirs.

**Figure 3:**
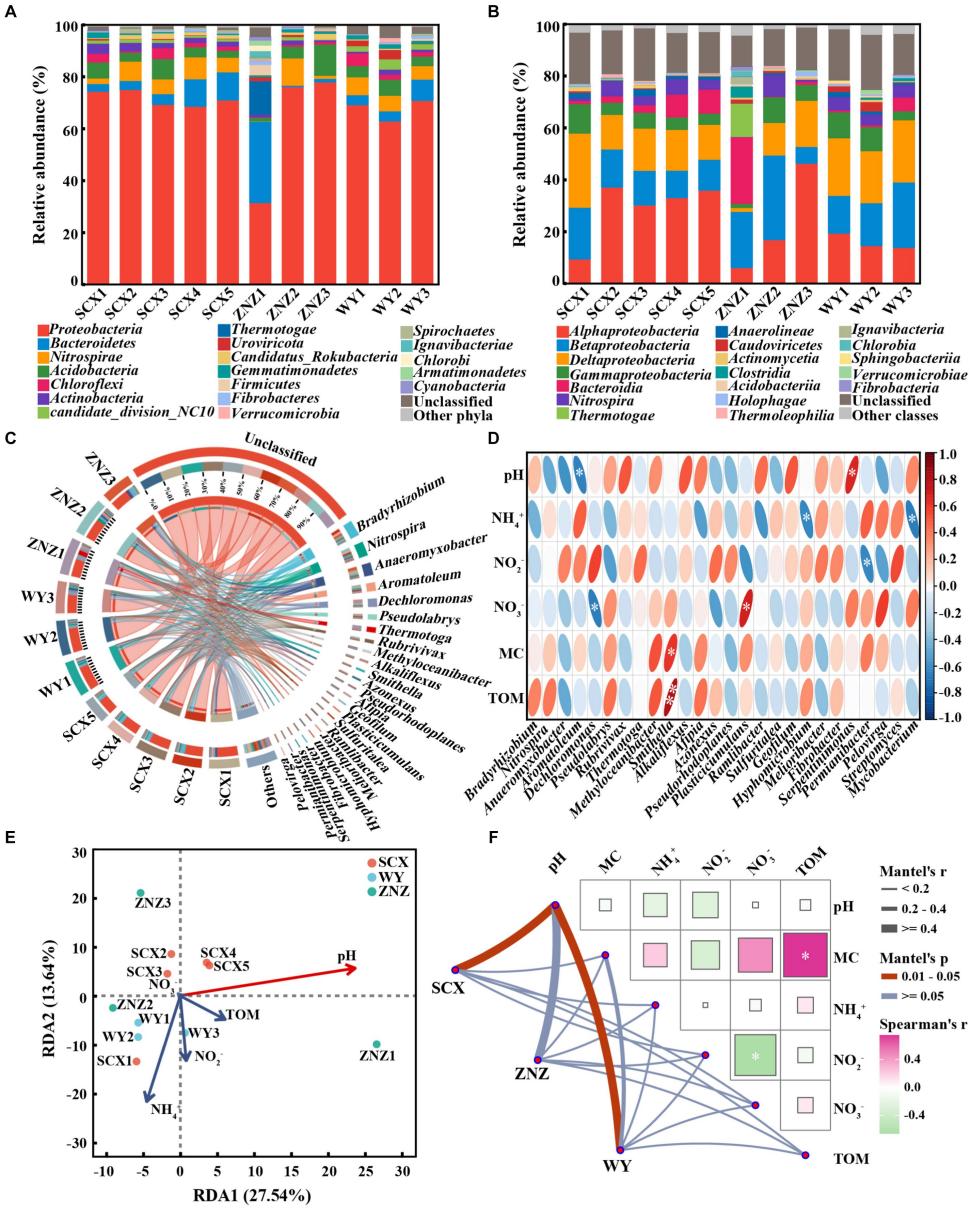
Taxonomic distribution of microbial communities harboring *rpf*-like genes, and their associated environmental factors. Distribution of phyla **(A)** and classes **(B)** with abundance greater than 1%. **(C)** Circos figures illustrating relationships between sediments and genera with abundance greater than 1%. **(D)** Spearman correlation analysis between environmental factors and dominant genera (with abundance greater than 1%) as well as *Streptomyces* and *Mycobacterium*. The red and blue ellipses indicate positive and negative correlations, respectively. Significant correlations are denoted by asterisks: *p < 0.05 and **p < 0.01. **(E)** Redundancy analysis (RDA) demonstrating the link between microbial communities containing *rpf*-like genes and physiochemical parameters of sediments. The variables significantly influencing microbial communities, selected by the forward selection procedure, are highlighted in red on the ordinations. **(F)** Mantel test based on Spearman rank correlation exhibiting the correlations of environmental factors with microbial communities and the correlations among different physiochemical properties of sediments. MC, moisture content; TOM, total organic matter. Significance is denoted by asterisk: *p < 0.05.

In order to examine the distribution patterns of microbial communities harboring *rpf*-like genes in the reservoir sediments, an investigation into the microbial composition linked to the presence of *rpf*-like genes was carried out. At the phylum level (Figure 3A), *Proteobacteria* (31.56–78.04%) was the primary phylum harboring *rpf*-like genes in the reservoir sediments. *Bacteroidetes*, the second most abundant phylum containing *rpf*-like genes, exhibited an increased abundance from the upstream to downstream sections in the SCX and WY reservoirs. Remarkably, the presence of *Bacteroidetes* with *rpf*-like genes was significantly higher in the ZNZ1 (31.36%) compared to other sediment samples (0.51–10.81%). A range of 0.68 to 3.91% of *Actinobacteria* were also found to contain *rpf*-like genes, with the highest proportion detected in SCX1. Extensive documentation exists regarding the presence of *rpf*-like genes in high G + C Gram-positive *Actinobacteria* (Mukamolova et al., 2006; Shi et al., 2024b). At the class level (Figure 3B), *Alphaproteobacteria* (6.14–46.37%), a subgroup of the *Proteobacteria*, was the predominant class hosting *rpf*-like genes in the reservoir sediments, followed by *Betaproteobacteria* (6.47–32.62%). Additionally, the abundance of *Bacteroidia* (25.68%) containing *rpf*-like genes was notably elevated in the ZNZ1 in comparison to the other sediment samples, consistent with the findings from the phylum analysis. These results revealed that *rpf*-like genes in the three reservoirs were predominantly found in *Proteobacteria*, specifically in the *Alphaproteobacteria* and *Betaproteobacteria*.

At the genus level (Figure 3C), most of the genera containing *rpf*-like genes in the reservoir sediments were categorized as unclassified. In the SCX reservoir, *Bradyrhizobium* (5.24–10.20%) was the predominant genus, except for SCX1 where *Anaeromyxobacter* was dominant. Significant differences were observed in the distribution of genera containing *rpf*-like genes from the upstream to downstream sections of the ZNZ reservoir. The predominant genera were *Thermotoga*, *Nitrospira*, and *Anaeromyxobacter* in ZNZ1, ZNZ2, and ZNZ3, respectively. In the WY reservoir, *Nitrospira* (4.20–5.17%) was the dominant genus, except for WY3 where *Dechloromonas* was prevalent. It is noteworthy that genera harboring *rpf*-like genes, such as *Bradyrhizobium*, *Anaeromyxobacter*, and *Nitrospira*, have been extensively reported for their involvement in nitrogen cycling (Wang et al., 2021; Sun et al., 2023). Although several studies have reported the presence of *rpf*-like genes in *Streptomyces* and *Mycobacterium* (Mukamolova et al., 2006; Kana et al., 2008), *Streptomyces* with *rpf*-like genes were only found in SCX1 (0.08%) and SCX2 (0.33%), and *Mycobacterium* with *rpf*-like genes were only detected in SCX5 (0.08%) and ZNZ3 (0.13%).

The association between genera harboring *rpf*-like genes and environmental factors was examined through heat map analysis (Figure 3D). The relative abundance of *Aromatoleum* exhibited a significant (*p* < 0.05) and negative correlation with pH, while *Dechloromonas* showed a significant (*p* < 0.05) and negative correlation with NO_3_^−^ concentration. Similarly, the relative abundances of *Hyphomicrobium* and *Mycobacterium* displayed significant (*p* < 0.05) and negative correlations with NH_4_^+^ concentration. The relative abundance of *Permianibacter* was significantly (*p* < 0.05) and negatively correlated with NO_2_^−^ concentration. In contrast, the relative abundance of *Smithella* was significantly and positively correlated with MC (*p* < 0.05) and TOM (*p* < 0.01). The relative abundance of *Plasticicumulans* exhibited a significant (*p* < 0.05) and positive correlation with NO_3_^−^, while the relative abundance of *Serpentinimonas* showed a significant (*p* < 0.05) and positive correlation with pH. The heat map analysis revealed that environmental factors had diverse effects on species harboring *rpf*-like genes, thus influencing the microbial community structure. Furthermore, by integrating local environmental variables with community structures, the correlation between environmental factors and community compositions containing *rpf*-like genes was identified. As illustrated in Figure 3E, the first two components explained 41.18% of the microbial diversity containing *rpf*-like genes. The RDA results supported the significant influence of pH (*p* < 0.05) on the distribution of microbial communities with *rpf*-like genes in the reservoir sediments. Additionally, pairwise Spearman’s correlation and partial Mantel tests were further employed to reveal the environmental drivers of communities harboring *rpf*-like genes in the three reservoirs (Figure 3F). The microbial communities containing *rpf*-like genes in SCX and WY reservoirs were significantly (*r* = 0.58, *p* < 0.05; *r* = 0.52, *p* < 0.05) correlated with pH, indicating that pH exerted a primary influence on the species compositions containing *rpf*-like genes.

### 3.3 Microbial community and functional genes involved in nitrogen cycling and their correlation with environmental factors

Based on the KEGG annotation, nitrogen-cycling functional genes associated with nitrogen fixation, nitrification, denitrification, DNRA, ANRA, anammox, and ammonification were annotated in all sediment samples (Supplementary Figure S3 and Supplementary Table S4). Denitrification was found to be the predominant function, constituting 34.67% of the total nitrogen transformation functions (Supplementary Figure S4). Ammonification and DNRA genes accounted for 18.16 and 16.73% of the total nitrogen transformation functions, respectively, while nitrogen fixation genes exhibited the lowest proportion at 7.23%. Compared to denitrification, the DNRA process prefers highly reduced environments (Zhang et al., 2023c). The dominance of denitrification over DNRA in the reservoir sediments may be attributed to the less reduced environments. Notably, the key genes, *hzsA*, *hzsB*, and *hdh*, known to be involved in anammox (Kuypers et al., 2018; Wang et al., 2022a), were absent in all samples. This absence could be explained by the low tolerance of anammox bacteria to oxygen, which inhibits their activity even under low oxygen levels. Molecular oxygen can directly poison redox enzymes, particularly Fe-S proteins with low redox potentials (such as ferredoxins and hydrogenases), essential for electron transfer. Furthermore, during the oxygen reduction process, intracellular reactive oxygen species are generated due to electron leakage, leading to oxidative stress that further hampers the activity of anammox bacteria (Zhang et al., 2023b).

**Figure 4:**
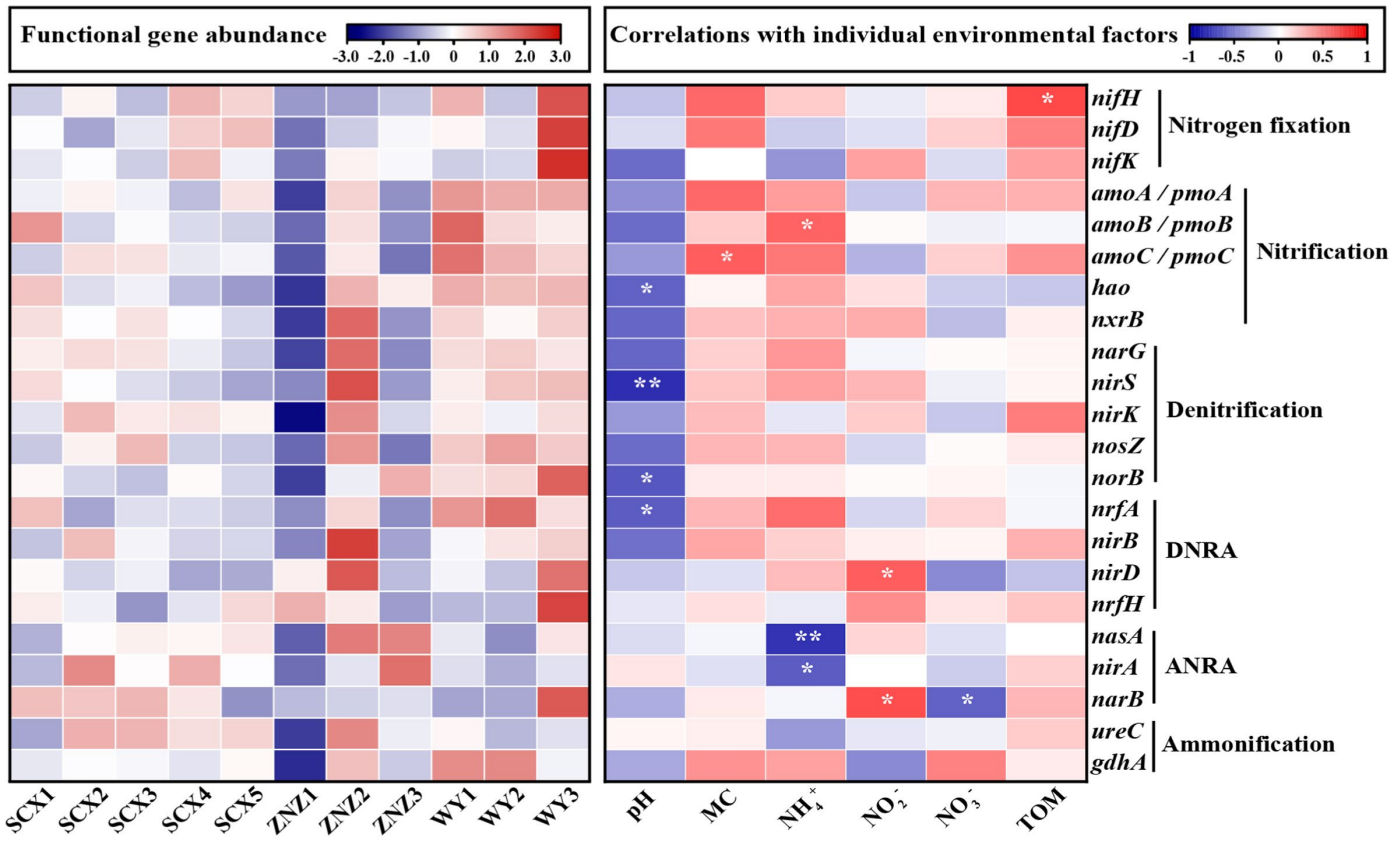
Heatmap depicting the abundance of functional genes related to the nitrogen cycle in reservoir sediment samples (left plot), with high abundance represented in red and low abundance in blue. The abundance of functional genes was normalized using row scaling. The right plot illustrates the correlations (positive in red, negative in blue) between nitrogen-cycling genes and environmental factors. MC, moisture content; TOM, total organic matter. Significance levels are indicated by asterisks: *p < 0.05 and **p < 0.01.

The heatmap illustrating the correlations between nitrogen-cycling genes and environmental factors is presented in Figure 4. Nitrogen fixation genes were predominantly located downstream of the WY reservoir, particularly in WY3, with a minor distribution observed in the downstream sections of SCX4 and SCX5 in the SCX reservoir. The abundance of *nifH*, involved in nitrogen fixation, demonstrated a significant positive correlation with the TOM. The relatively higher abundance of *nifH* in the downstream section may be attributed to reduced flow, resulting in TOM deposition (Wakelin et al., 2008). Nitrification genes were predominantly present in the WY reservoir, with the *amoB* gene, responsible for ammonia oxidation, exhibiting a significant positive correlation with NH_4_^+^ concentration, a relationship previously observed by Hallam et al. (2006). Denitrification and DNRA genes were mainly distributed in the ZNZ2, with a high distribution also observed in the WY reservoir. The abundance of *nirS* and *norB* genes was significantly associated with pH, while the fluctuation in *nirD* corresponded to that of NO_2_^−^ concentration. Furthermore, ANRA genes were primarily distributed downstream of the ZNZ reservoir, with significant negative correlations observed between the abundance of *nasA* and *nirA* genes and NH_4_^+^ concentration. The *narB* gene exhibited significant correlations with NO_3_^−^ and NO_2_^−^ concentrations. Additionally, the abundance of ammonification genes in the three reservoirs displayed a decreasing trend from the upstream to downstream sections.

**Figure 5:**
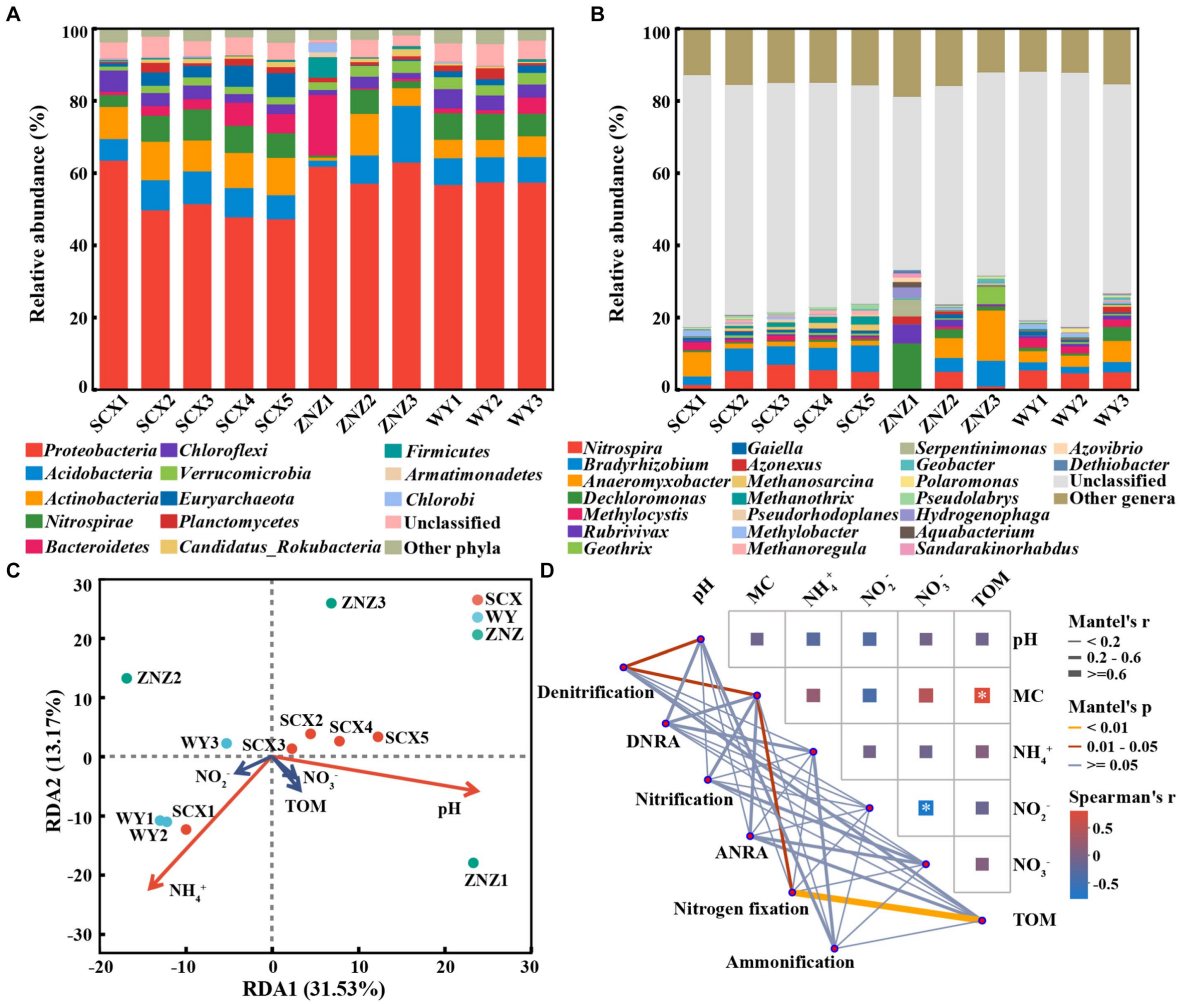
Taxonomic distribution of nitrogen-cycling functional microorganisms in reservoir sediments, and their associated environmental factors. Distribution of phyla **(A)** and genera **(B)** with abundance greater than 1%. **(C)** Redundancy analysis (RDA) illustrating the relationship between microbial communities hosting nitrogen-cycling functional genes and sediment physiochemical parameters. Variables significantly influencing microbial communities, identified through a forward selection procedure, are highlighted in red. **(D)** Mantel test, based on Spearman rank correlation, demonstrating the correlations of environmental factors with microbial communities involved in the six major nitrogen cycling processes in sediments, as well as the correlations among different physiochemical properties of the sediments. Significance is denoted by asterisk: *p < 0.05.

Following taxonomic assignment, a total of 617 genera within 61 phyla containing nitrogen-cycling genes were identified in all sediment samples. At the phylum level (Figure 5A), the majority of nitrogen-cycling functional genes were associated with *Proteobacteria* (47.40–63.66%), followed by *Acidobacteria* (1.64–15.67%), *Actinobacteria* (0.84–11.49%), *Nitrospirae* (0.62–8.58%), *Bacteroidetes* (0.40–16.72%), and *Chloroflexi* (1.40–5.84%). Notably, the abundance of *Bacteroidetes* in ZNZ1 was significantly higher compared to the other sediments. At the genus level (Figure 5B), apart from unclassified genera, 23 genera with relative abundance greater than 1% were detected, with *Nitrospira* (0.07–7.07%), *Bradyrhizobium* (0.34–7.32%), *Anaeromyxobacter* (0.03–13.93%), and *Dechloromonas* (0.30–12.53%) being the most dominant. To elucidate the primary chemical characteristics of sediment supporting the nitrogen-cycling microbial community, RDA was conducted to assess the sediment chemical properties exerting the most significant influence. RDA1 and RDA2 could explain 44.70% of the total variation (Figure 5C), with SCX and WY samples exhibiting greater clustering, while ZNZ samples appeared more dispersed. The composition of sediment nitrogen-cycling microbial communities in the SCX, ZNZ, and WY exhibited significant correlations with NH_4_^+^ (*p* < 0.01) and pH (*p* < 0.05). Furthermore, partial Mantel tests were employed to further elucidate the environmental drivers of nitrogen-cycling microbial communities in the sediments (Figure 5D). The community structure of denitrifiers was significantly influenced by pH (*r* = 0.41, *p* < 0.05) and MC (*r* = 0.42, *p* < 0.05), while the nitrogen-fixing microbial community was primarily affected by TOM (*r* = 0.61, *p* < 0.01). Figure 5D also illustrated the correlations among different sediment parameters, with the exclusion of high collinearity (|*r*| > 0.8) (Ding et al., 2022). Specifically, TOM was found to be significantly correlated with MC (*r* = 0.75, *p* < 0.05). The results suggested that TOM and MC may jointly or independently impact the composition of nitrogen-fixing microbial communities (Zhang et al., 2023a).

**Figure 6:**
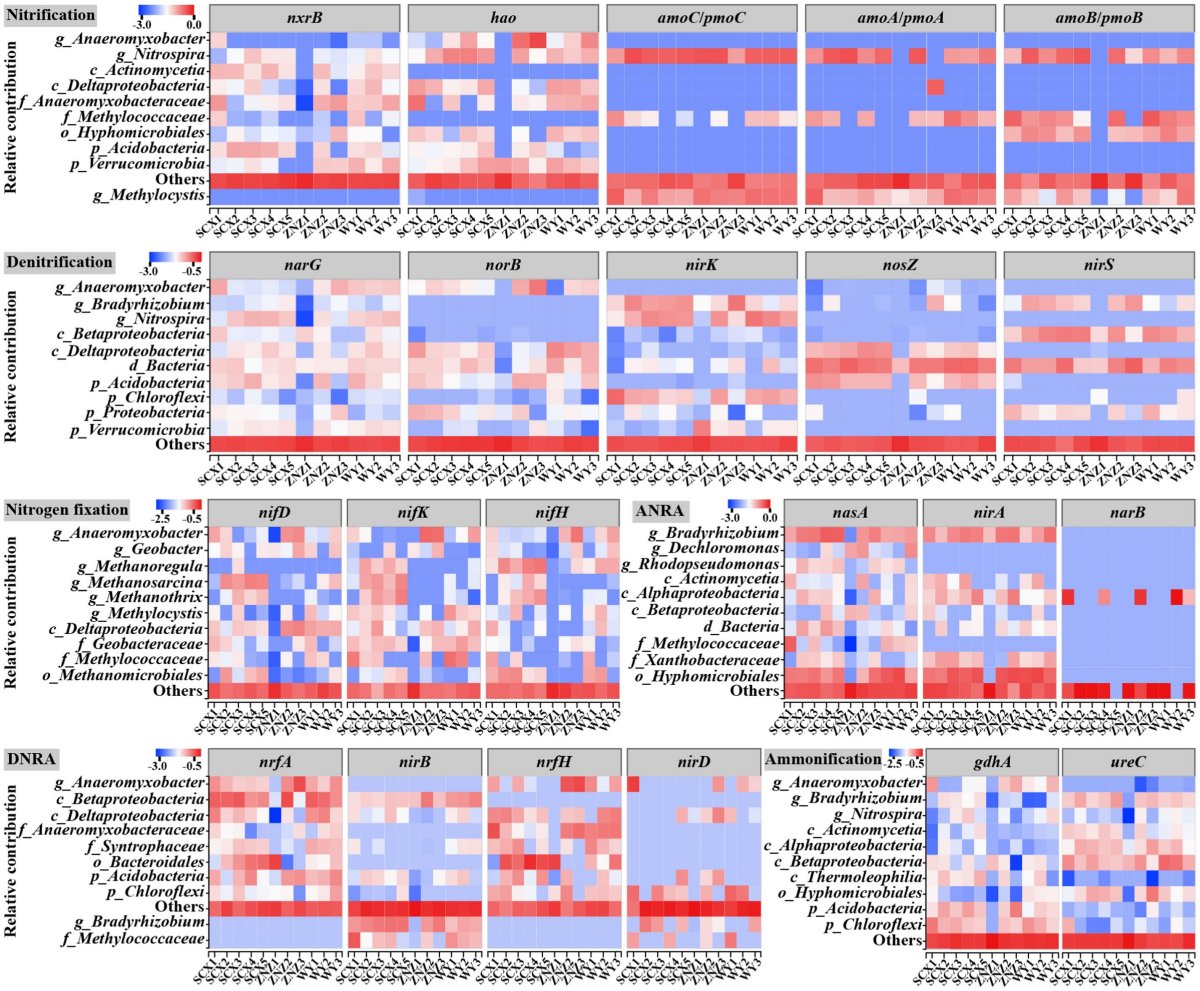
Taxonomic contributions to nitrogen-cycling functional genes across various nitrogen transformation processes in diverse water reservoir sediment samples.

Additionally, the relative contributions of different taxa to nitrogen-cycling genes across various nitrogen transformation processes were evaluated (Figure 6). The majority of nitrogen fixation genes were associated with *Anaeromyxobacter*, *Geobacter*, and *Methanoregula*. Notably, *Methanoregula* was found to harbor the *nifH* gene, which encodes iron-containing electron transfer proteins (Kuypers et al., 2018), and exhibited significant enrichment in downstream sites, regulating the downstream nitrogen-fixing process. Regarding the nitrification process, *amoA*, *amoB*, *amoC*, *hao*, and *nxrB* were predominantly assigned to the genus *Nitrospira*, known for its ability to oxidize ammonia in ammonia-limited environments (Kits et al., 2017). The prevalence of *Nitrospira* in the SCX reservoir suggested its potential involvement in the complete ammonia oxidation process. Furthermore, the main denitrifiers, namely, *Bradyrhizobium* and *Nitrospira*, exhibited higher abundance downstream of the reservoirs compared to upstream. *Bradyrhizobium*, also identified as the predominant genus in the ANRA process, was mainly associated with the abundance of *nasA*, encoding the catalytic subunit of assimilatory nitrate reductase (Hu et al., 2022), and demonstrated increased prevalence at downstream sites. Hidalgo-García et al. (2019) demonstrated the generation of nitrous oxide through the interplay of the ANRA and denitrification processes, indicating the promotion of the initial step of the ANRA process at downstream sites, subsequently providing more substrate for denitrification. Meanwhile, most of the ammonification genes were also assigned to the genus *Bradyrhizobium*, as well as *Anaeromyxobacter*. In the DNRA process, *nrfA* and *nrfH* were predominantly attributed to the genus *Anaeromyxobacter*, and unclassified genera in *Deltaproteobacteria* and *Anaeromyxobacteraceae*, while *nirB* was mainly assigned to unclassified genera in *Betaproteobacteria*. Previous studies have highlighted the role of DNRA in generating NH_4_^+^ to enhance primary productivity in oligotrophic marine and soil systems (Lam et al., 2009; Shan et al., 2016), a process that may also occur in water reservoir sediments.

### 3.4 Co-occurrence of rpf-like genes with genes associated with nitrogen cycle and VBNC formation

**Figure 7:**
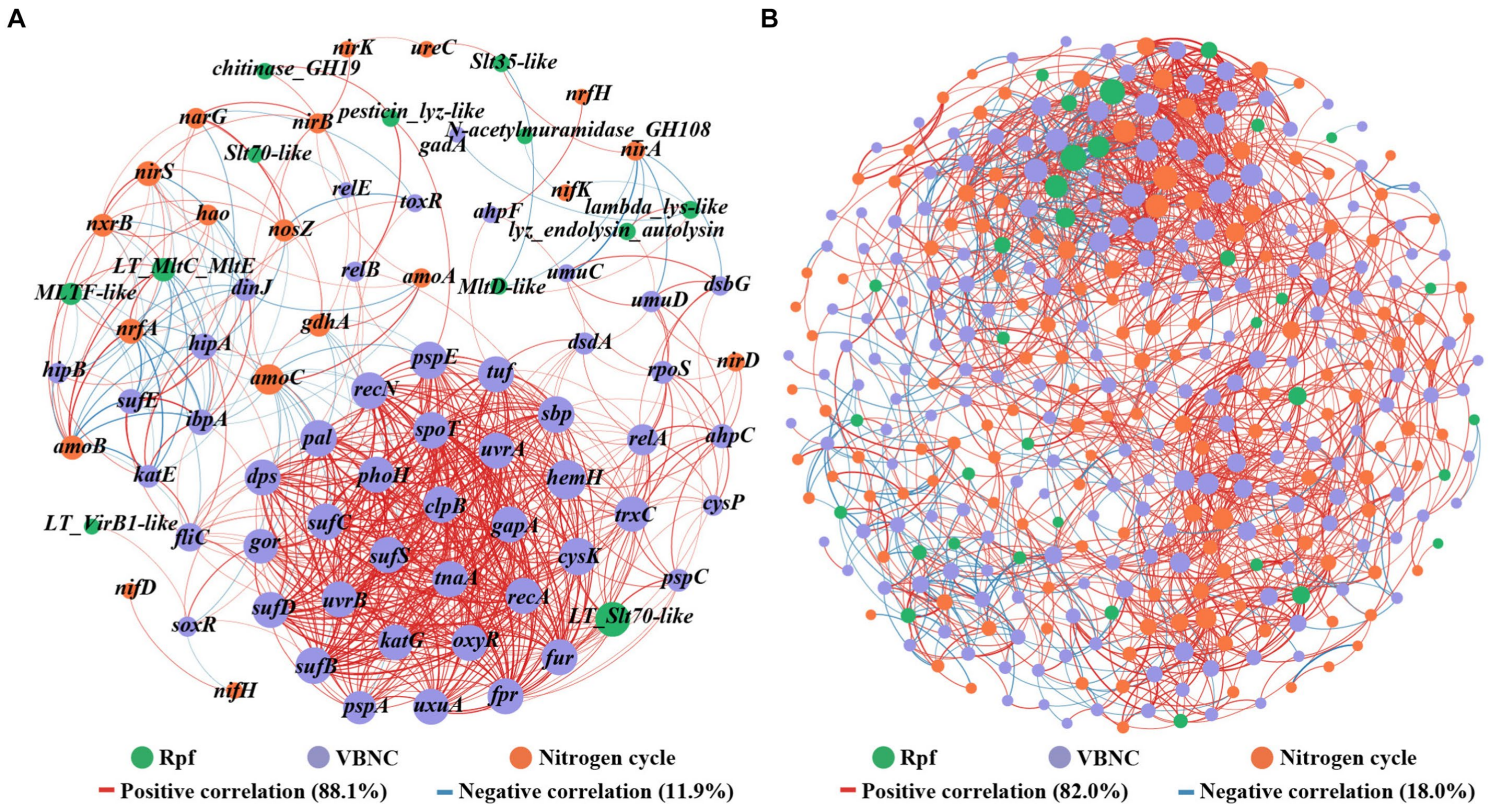
Co-occurrence patterns among *rpf*-like genes, nitrogen-cycling functional genes, and VBNC-related genes **(A)**, as well as among associated genera **(B)**. Links represent statistically significant associations between nodes (|*r*| ≥ 0.7, *p* < 0.01). The size of each node is proportional to the number of connections. The red link indicates a positive correlation, and the blue link indicates a negative correlation. The thickness of each connection between two nodes (i.e., edge) is proportional to the strength of the correlation.

The network analysis based on Spearman correlation revealed co-occurrence patterns among *rpf*-like genes, nitrogen-cycling functional genes, and VBNC-related genes with strong correlations (Figure 7A). The network consisting of 81 nodes connected by 638 edges, with 88.1% positive and 11.9% negative interactions. Notably, the *rpf*-like genes demonstrated 11 positive connections and 4 negative connections with nitrogen-cycling functional genes. Among these, the *MLTF*-like gene, which showed high abundance in all three reservoirs, displayed the highest number of connections with nitrogen-cycling functional genes and primarily positive correlations, indicating its crucial role in the positive correlation between *rpf*-like genes and nitrogen-cycling functional genes. Furthermore, the *rpf*-like genes demonstrated 33 positive links and 13 negative links with VBNC-related genes, with the *LT_Slt70-like* gene specifically linked to VBNC-related genes through 28 positive connections. Additionally, VBNC-related genes showed the highest self-co-occurrence rate, comprising around 74.5% of the occurrences.

To unravel the intricate microbial co-occurrence network, correlation network analysis was conducted on the gene-associated genera. As depicted in Figure 7B, the network consisted of 364 nodes connected by 1874 edges, with 82.0% representing positive correlations and 18.0% reflecting negative correlations among these genera. A modularity index exceeding 0.4 is widely acknowledged as indicative of a modular structure (Jiao et al., 2016), and the observed modularity index of 0.58 suggested a modular construction of functional microbial populations in the water reservoir sediments. Within the network, the key genera *Pseudolabrys*, *Bradyrhizobium*, *Nitrospira*, *Anaeromyxobacter*, and *Rubrivivax*, which all contained nitrogen-cycling functional genes, *rpf*-like genes, and VBNC-related genes, collectively constituted 5.4, 4.9, 3.3, 2.2, and 1.7% of the network, respectively. Previous studies have documented the participation of *Pseudolabrys* in denitrification processes (Kämpfer et al., 2006; Li et al., 2022). Notably, *Pseudolabrys* appeared to be a significant genus within the network, possessing all three types of genes, with a higher degree (degree: 105) and betweenness centrality. *Dechloromonas* was noted for harboring both nitrogen-cycling functional genes and *rpf*-like genes. Furthermore, 11 genera were found to contain nitrogen-cycling functional genes and VBNC-related genes (Supplementary Table S8), suggesting the possibility of microorganisms involved in the nitrogen cycle entering the VBNC state. The results indicated that these nitrogen-cycling functional microbial populations included microorganisms with *rpf*-like genes and VBNC-related genes. Overall, the interaction between *rpf*-like genes and genes involved in nitrogen cycle and VBNC formation, along with their associated genera, revealed a strong correlation of Rpf homologs with functional microbial communities in water reservoir sediments.

## 4 Conclusion

The present study demonstrated the distinct microbial communities and functional populations present in the water reservoir sediments. Importantly, it revealed the widespread occurrence of Rpf-like domains and related genes across the three reservoir sediments. The *rpf*-like genes were primarily associated with *Bradyrhizobium*, *Nitrospira*, and *Anaeromyxobacter*. The distribution of *rpf*-like genes and their related genera in the sediments showed correlations with various environmental factors, with pH emerging as the primary influential factor. Furthermore, denitrification genes were identified as the primary nitrogen-cycling functional genes in the reservoir sediments, followed by ammonification and DNRA genes, with nitrogen fixation genes were comparatively less abundant. The majority of nitrogen-cycling functional genes were found in *Bradyrhizobium* and *Dechloromonas* within *Proteobacteria*, as well as the genera *Nitrospira* and *Anaeromyxobacter*. The presence of nitrogen-cycling microbial communities in the reservoir sediments was predominantly influenced by pH and NH_4_^+^. Notably, a strong correlation was observed between *rpf*-like genes and genes associated with the nitrogen cycle and VBNC formation, indicating the potential of Rpf homologs in resuscitating and stimulating functional microbial populations in the water reservoir sediments. This study provided novel insights into the prevalence of *rpf*-like genes in water reservoir sediments and advanced our understanding of the relationship between microorganisms containing *rpf*-like genes and nitrogen-cycling functional populations, offering new perspectives for the huge potential of microbial nitrogen cycling in water reservoirs.

## Data availability statement

The datasets presented in this study can be found in online repositories. The names of the repository/repositories and accession number(s) can be found in the article/Supplementary material.

## Author contributions

AH: Data curation, Investigation, Writing – original draft. HF: Data curation, Investigation, Writing – original draft. LL: Investigation, Resources, Writing – review & editing. XS: Conceptualization, Project administration, Supervision, Writing – original draft, Writing – review & editing. SZ: Project administration, Supervision, Writing – original draft, Writing – review & editing. JL: Resources, Software, Writing – review & editing. FS: Resources, Writing – review & editing.

## Footnotes

^1^ https://www.ncbi.nlm.nih.gov/Structure/cdd/cdd.shtml
