# Supplementary material for: Exploring the distribution and co-occurrence of rpf-like genes and nitrogen-cycling genes in water reservoir sediments
Source: Front Microbiol. 2024 Jul 22;15:1433046. doi: 10.3389/fmicb.2024.1433046 (PMC11298755; doi:10.3389/fmicb.2024.1433046)
Supplement: Supplementary file 1 [file Table_1.DOCX]

**Supplemental Material**

**Exploring the distribution and co-occurrence of *rpf*-like genes and nitrogen-cycling genes in water reservoir sediments**

Aiqin Hou ^a, 1^, Huayi Fu^a, 1^, Leilei Liu^b^, Xiaomei Su^a,^*, Shusheng Zhang^b,^*, Jiahou Lai^b^, Faqian Sun^a^

^a^College of Geography and Environmental Science, Zhejiang Normal University, Jinhua 321004, China

^b^The Management Center of Wuyanling National Natural Reserve in Zhejiang, Wenzhou 325500, China

^1^These authors contributed equally to this work.

*Corresponding author: Xiaomei Su, Shusheng Zhang

Address: Yingbin Road 688#, Jinhua, 321004, China

E-mail: [purple@zjnu.cn](mailto:purple@zjnu.cn); [zyhx2658@163.com](mailto:zyhx2658@163.com)

**Table S1** Physical and chemical properties of the sediment samples from water reservoirs. The sampling sites Sanchaxi, Zhangnenzi, and Wenyang reservoirs are denoted by SCX, ZNZ, and WY, respectively. MC represents moisture content; NH_4_^+^ denotes ammonium; NO_2_^-^ indicates nitrite; NO_3_^-^ represents nitrate; and TOM stands for total organic matter. Different letters following the values in the same column indicate a significant difference among the sampling sites (Tukey's test; *P* < 0.05).

| **Number** | **pH** | **MC**  **(%)** | **NH_4_^+^**  **(mg kg^-1^)** | **NO_2_^-^**  **(mg kg^-1^)** | **NO_3_^-^**  **(mg kg^-1^)** | **TOM**  **(%)** |
| --- | --- | --- | --- | --- | --- | --- |
| SCX1 | 7.03 | 54.45±0.55 e | 17.30±0.17 b | 0.29±0.01 a | 0.00 | 5.74±0.24 b |
| SCX2 | 7.27 | 57.95±1.18 cde | 16.90±0.67 b | 0.22±0.01 bc | 0.00 | 9.59±0.34 b |
| SCX3 | 7.48 | 61.48±1.10 bc | 13.50±1.44 c | 0.16±0.00 e | 0.00 | 8.59±0.27 b |
| SCX4 | 7.09 | 56.72±4.38 de | 8.03±0.54 d | 0.16±0.00 e | 0.00 | 10.81±1.37 b |
| SCX5 | 7.70 | 63.94±1.04 b | 6.43±0.33 d | 0.14±0.00 fg | 0.23±0.17 | 8.99±0.30 a |
| ZNZ1 | 8.51 | 53.93±1.47 e | 14.63±0.34 c | 0.21±0.00 c | 0.00 | 6.78±3.64 b |
| ZNZ2 | 6.80 | 40.08±1.42 f | 7.83±0.19 d | 0.22±0.00 bc | 0.00 | 4.23±0.09 b |
| ZNZ3 | 7.34 | 33.54±1.99 g | 5.93±1.01 d | 0.18±0.00 d | 0.00 | 4.00±0.51 b |
| WY1 | 7.41 | 58.26±0.32 cde | 26.10±1.31 a | 0.15±0.01 ef | 0.00 | 8.08±0.13 b |
| WY2 | 6.26 | 59.73±1.00 bcd | 25.80±1.73 a | 0.13±0.00 g | 0.17±0.09 | 7.65±0.01 a |
| WY3 | 6.42 | 71.39±0.72 a | 14.50±0.42 c | 0.23±0.01 b | 0.00 | 15.14±0.42 b |

**Table S2** Physical and chemical properties of the water samples from water reservoirs. The sampling sites Sanchaxi, Zhangnenzi, and Wenyang reservoirs are denoted by SCX, ZNZ, and WY, respectively. NH_4_^+^ denotes ammonium; NO_2_^-^ indicates nitrite; NO_3_^-^ represents nitrate; TP stands for total phosphorus; TOC represents total organic carbon; and TN stands for total nitrogen.

| **Number** | **pH** | **NH_4_^+^**  **(mg L^-1^)** | **NO_2_^-^**  **(mg L^-1^)** | **NO_3_^-^**  **(mg L^-1^)** | **TP**  **(mg L^-1^)** | **TOC**  **(mg L^-1^)** | **TN**  **(mg L^-1^)** |
| --- | --- | --- | --- | --- | --- | --- | --- |
| SCX1 | 7.52 | 0.07±0.01 | 0.00 | 0.17±0.12 | 0.00 | 2.23±0.11 | 0.68±0.03 |
| SCX2 | 7.06 | 0.03±0.04 | 0.00 | 0.00 | 0.00 | 3.10±0.13 | 0.08±0.04 |
| SCX3 | 7.66 | 0.00 | 0.00 | 0.00 | 0.01±0.01 | 2.86±0.20 | 0.10±0.03 |
| SCX4 | 7.49 | 0.00 | 0.00 | 0.00 | 0.00 | 2.95±0.06 | 0.05±0.05 |
| SCX5 | 7.56 | 0.00 | 0.00 | 0.00 | 0.01±0.01 | 3.51±0.13 | 0.08±0.05 |
| ZNZ1 | 7.84 | 0.01±0.01 | 0.00 | 0.93±0.05 | 0.00 | 1.82±0.06 | 1.06±0.20 |
| ZNZ2 | 7.62 | 0.00 | 0.00 | 0.20±0.28 | 0.00 | 2.55±0.07 | 0.21±0.10 |
| ZNZ3 | 7.66 | 0.00 | 0.00 | 0.00 | 0.00 | 1.79±0.01 | 0.06±0.00 |
| WY1 | 7.76 | 0.05±0.03 | 0.00 | 0.13±0.09 | 0.01±0.01 | 1.92±0.06 | 0.20±0.03 |
| WY2 | 7.57 | 0.00 | 0.00 | 0.00 | 0.00 | 3.06±0.08 | 0.10±0.02 |
| WY3 | 7.34 | 0.01±0.01 | 0.03±0.00 | 0.03±0.05 | 0.00 | 0.63±0.01 | 0.13±0.02 |

**Table S3** The distribution of Rpf-like domains and associated genes in sediment samples from water reservoirs. The sampling sites Sanchaxi, Zhangnenzi, and Wenyang reservoirs are denoted by SCX, ZNZ, and WY, respectively.

| **Rpf-like domains** | **Description** | **SCX1** | **SCX2** | **SCX3** | **SCX4** | **SCX5** | **ZNZ1** | **ZNZ2** | **ZNZ3** | **WY1** | **WY2** | **WY3** |
| --- | --- | --- | --- | --- | --- | --- | --- | --- | --- | --- | --- | --- |
| cd13402 | LT_TF-like | 0 | 0 | 0 | 0 | 0 | 1 | 0 | 0 | 0 | 0 | 0 |
| cd00325 | chitinase_GH19 | 1 | 0 | 0 | 0 | 0 | 6 | 2 | 1 | 1 | 0 | 0 |
| cd00737 | lyz_endolysin_autolysin | 6 | 0 | 1 | 0 | 0 | 68 | 3 | 3 | 5 | 12 | 4 |
| cd00978 | chitosanase_GH46 | 0 | 0 | 0 | 0 | 0 | 3 | 1 | 2 | 0 | 0 | 1 |
| cd16904 | pesticin_lyz-like | 2 | 0 | 0 | 0 | 0 | 8 | 1 | 1 | 0 | 0 | 0 |
| cd13926 | N-acetylmuramidase_GH108 | 2 | 2 | 0 | 0 | 0 | 32 | 1 | 2 | 3 | 7 | 0 |
| cd00736 | lambda_lys-like | 1 | 0 | 1 | 0 | 0 | 9 | 0 | 0 | 1 | 1 | 1 |
| cd00254 | LT-like | 52 | 16 | 7 | 17 | 12 | 137 | 44 | 72 | 6 | 7 | 29 |
| cd13399 | Slt35-like | 23 | 21 | 6 | 11 | 11 | 60 | 23 | 51 | 3 | 5 | 21 |
| cd13400 | LT_IagB-like | 2 | 2 | 0 | 0 | 0 | 21 | 3 | 0 | 0 | 1 | 3 |
| cd13401 | Slt70-like | 61 | 13 | 11 | 11 | 17 | 122 | 48 | 73 | 7 | 9 | 33 |
| cd13403 | MLTF-like | 61 | 12 | 9 | 9 | 5 | 48 | 42 | 15 | 9 | 17 | 30 |
| cd16892 | LT_VirB1-like | 1 | 0 | 0 | 0 | 0 | 9 | 2 | 2 | 1 | 0 | 0 |
| cd16893 | LT_MltC_MltE | 7 | 0 | 0 | 0 | 3 | 4 | 5 | 1 | 0 | 0 | 3 |
| cd16896 | LT_Slt70-like | 30 | 4 | 3 | 1 | 5 | 50 | 13 | 29 | 4 | 3 | 11 |
| cd16894 | MltD-like | 67 | 15 | 6 | 13 | 17 | 140 | 36 | 51 | 17 | 21 | 51 |
| cd00442 | Lyz-like | 0 | 0 | 0 | 0 | 0 | 1 | 0 | 0 | 0 | 0 | 0 |

# **Table S4** Nitrogen-cycling functional genes and related KEGG Orthology (KO).

| **KO ID** | **KO Description**  **[EC number]** | **Gene** |
| --- | --- | --- |
| **Nitrogen fixation** |  |  |
| K02588 | nitrogenase iron protein NifH | *nifH* |
| K02586 | nitrogenase molybdenum-iron protein alpha chain [EC:1.18.6.1] | *nifD* |
| K02591 | nitrogenase molybdenum-iron protein beta chain [EC:1.18.6.1] | *nifK* |
| **Nitrification** |  |  |
| K10944 | methane/ammonia monooxygenase subunit A [EC:1.14.18.3 1.14.99.39] | *amoA/pmoA* |
| K10945 | methane/ammonia monooxygenase subunit B | *amoB/pmoB* |
| K10946 | methane/ammonia monooxygenase subunit C | *amoC/pmoC* |
| K10535 | hydroxylamine dehydrogenase [EC:1.7.2.6] | *hao* |
| K00371 | nitrate reductase/nitrite oxidoreductase, beta subunit [EC:1.7.5.1 1.7.99.-] | *nxrB* |
| **Denitrification** |  |  |
| K00370 | nitrate reductase/nitrite oxidoreductase, alpha subunit [EC:1.7.5.1 1.7.99.-] | *narG* |
| K15864 | nitrite reductase (NO-forming)/hydroxylamine reductase [EC:1.7.2.1 1.7.99.1] | *nirS* |
| K00368 | nitrite reductase (NO-forming) [EC:1.7.2.1] | *nirK* |
| K00376 | nitrous-oxide reductase [EC:1.7.2.4] | *nosZ* |
| K04561 | nitric oxide reductase subunit B [EC:1.7.2.5] | *norB* |
| **Dissimilatory nitrite reduction to ammonium (DNRA)** |  |  |
| K03385 | nitrite reductase (cytochrome c-552) [EC:1.7.2.2] | *nrfA* |
| K00362 | nitrite reductase (NADH) large subunit [EC:1.7.1.15] | *nirB* |
| K00363 | nitrite reductase (NADH) small subunit [EC:1.7.1.15] | *nirD* |
| K15876 | cytochrome c nitrite reductase small subunit | *nrfH* |
| **Assimilatory nitrate reduction to ammonium (ANRA)** |  |  |
| K00372 | assimilatory nitrate reductase catalytic subunit [EC:1.7.99.-] | *nasA* |
| K00366 | ferredoxin-nitrite reductase [EC:1.7.7.1] | *nirA* |
| K00367 | ferredoxin-nitrate reductase [EC:1.7.7.2] | *narB* |
| **Anaerobic ammonium oxidation（Anammox）** |  |  |
| K20934 | hydrazine synthase subunit [EC:1.7.2.7] | *hzsA_1/hzsA_2* |
| K20935 | hydrazine dehydrogenase [EC:1.7.2.8] | *hdh/hzo* |
| K20933 | hydrazine synthase subunit [EC:1.7.2.7] | *hzsB* |
| K20932 | hydrazine synthase subunit [EC:1.7.2.7] | *hzsC* |
| **Ammonification** |  |  |
| K01428 | urease subunit alpha [EC:3.5.1.5] | *ureC* |
| K00261 | glutamate dehydrogenase (NAD(P)+) [EC:1.4.1.3] | *gdhA* |

**Table S5** VBNC-related genes detected in sediment samples from water reservoirs.

| **KO ID** | **KO Description**  **[EC number]** | **Gene** | **Reference** |
| --- | --- | --- | --- |
| K03695 | ATP-dependent Clp protease ATP-binding subunit ClpB | *clpB* | ([Dong et al., 2020](#_ENREF_4)) |
| K15773 | HTH-type transcriptional regulator / antitoxin HipB | *hipB* |  |
| K07473 | DNA-damage-inducible protein J | *dinJ* | ([Wood, 2016](#_ENREF_20)) |
| K04047 | starvation-inducible DNA-binding protein | *dps* | ([Liao et al., 2021](#_ENREF_11)) |
| K00134 | glyceraldehyde 3-phosphate dehydrogenase (phosphorylating) [EC:1.2.1.12] | *gapA* | ([Bai et al., 2022](#_ENREF_1)) |
| K03553 | recombination protein RecA | *recA* |  |
| K02358 | elongation factor Tu | *tuf* |  |
| K04761 | LysR family transcriptional regulator, hydrogen peroxide-inducible genes activator | *oxyR* | ([Wang et al., 2013](#_ENREF_19)) |
| K01139 | GTP diphosphokinase / guanosine-3',5'-bis(diphosphate) 3'-diphosphatase [EC:2.7.6.5 3.1.7.2] | *spoT* | ([Zhang et al., 2020](#_ENREF_22)) |
| K00951 | GTP pyrophosphokinase [EC:2.7.6.5] | *relA* |  |
| K03973 | phage shock protein C | *pspC* | ([Shi et al., 2024](#_ENREF_16)) |
| K03972 | phage shock protein E | *pspE* |  |
| K03812 | ribosome modulation factor | *rmf* |  |
| K03387 | NADH-dependent peroxiredoxin subunit F [EC:1.8.1.-] | *ahpF* | ([Zheng et al., 2001](#_ENREF_23)) |
| K03672 | thioredoxin 2 [EC:1.8.1.8] | *trxC* |  |
| K01738 | cysteine synthase [EC:2.5.1.47] | *cysK* |  |
| K01667 | tryptophanase [EC:4.1.99.1] | *tnaA* |  |
| K09013 | Fe-S cluster assembly ATP-binding protein | *sufC* |  |
| K11717 | cysteine desulfurase / selenocysteine lyase [EC:2.8.1.7 4.4.1.16] | *sufS* |  |
| K13255 | ferric iron reductase protein FhuF | *fhuF* |  |
| K06217 | phosphate starvation-inducible protein PhoH and related proteins | *phoH* |  |
| K02426 | cysteine desulfuration protein SufE | *sufE* |  |
| K23163 | sulfate/thiosulfate transport system substrate-binding protein | *sbp* |  |
| K01772 | protoporphyrin/coproporphyrin ferrochelatase [EC:4.98.1.1 4.99.1.9] | *hemH* |  |
| K04080 | molecular chaperone IbpA | *ibpA* |  |
| K09015 | Fe-S cluster assembly protein SufD | *sufD* |  |
| K00383 | glutathione reductase (NADPH) [EC:1.8.1.7] | *gor* |  |
| K09014 | Fe-S cluster assembly protein SufB | *sufB* |  |
| K03711 | Fur family transcriptional regulator, ferric uptake regulator | *fur* |  |
| K02048 | sulfate/thiosulfate transport system substrate-binding protein | *cysP* |  |
| K01753 | D-serine dehydratase [EC:4.3.1.18] | *dsdA* |  |
| K00528 | ferredoxin/flavodoxin---NADP+ reductase [EC:1.18.1.2 1.19.1.1] | *fpr* |  |
| K03805 | thiol:disulfide interchange protein DsbG | *dsbG* |  |
| K01686 | mannonate dehydratase [EC:4.2.1.8] | *uxuA* |  |
| K03503 | DNA polymerase V [EC:3.4.21.-] | *umuD* | ([Dubey et al., 2021](#_ENREF_5)) |
| K03631 | DNA repair protein RecN (Recombination protein N) | *recN* | ([Loke et al., 2016](#_ENREF_12)) |
| K03087 | RNA polymerase nonessential primary-like sigma factor | *rpoS* | ([Kusumoto et al., 2012](#_ENREF_7)) |
| K03970 | phage shock protein B | *pspB* | ([Loke et al., 2016](#_ENREF_12)) |
| K03781 | catalase [EC:1.11.1.6] | *katE* | ([Liao et al., 2019](#_ENREF_10)) |
| K03702 | excinuclease ABC subunit B | *uvrB* | ([Yu et al., 2022](#_ENREF_21)) |
| K07154 | serine/threonine-protein kinase HipA [EC:2.7.11.1] | *hipA* | ([Urbaniec et al., 2022](#_ENREF_18)) |
| K18918 | RHH-type transcriptional regulator, rel operon repressor / antitoxin RelB | *relB* | ([Pedersen et al., 2002](#_ENREF_15)) |
| K03701 | excinuclease ABC subunit A | *uvrA* | ([Kwan et al., 2015](#_ENREF_8)) |
| K10921 | cholera toxin transcriptional activator | *toxR* | ([Cai et al., 2023](#_ENREF_2)) |
| K03502 | DNA polymerase V | *umuC* | ([Papadimitriou et al., 2016](#_ENREF_14)) |
| K03969 | phage shock protein A | *pspA* | ([Li et al., 2024](#_ENREF_9)) |
| K02406 | flagellin | *fliC* | ([Gannon et al., 1997](#_ENREF_6)) |
| K03782 | catalase-peroxidase [EC:1.11.1.21] | *katG* | ([Chen et al., 2018](#_ENREF_3)) |
| K13639 | MerR family transcriptional regulator, redox-sensitive transcriptional activator SoxR | *soxR* |  |
| K01580 | glutamate decarboxylase [EC:4.1.1.15] | *gadA* |  |
| K03640 | peptidoglycan-associated lipoprotein | *pal* | ([Sulaiman et al., 2018](#_ENREF_17)) |
| K06218 | mRNA interferase RelE/StbE | *relE* | ([Zhu et al., 2013](#_ENREF_24)) |
| K24119 | NADH-dependent peroxiredoxin subunit C [EC:1.11.1.26] | *ahpC* | ([Wang et al., 2013](#_ENREF_19)) |
| K13053 | cell division inhibitor SulA | *sulA* | ([Mohiuddin Sayed et al., 2022](#_ENREF_13)) |

**Table S6** Information and quality of metagenome assembly of the sediment samples from water reservoirs.

| **Sample names** | **Raw reads**  **(M)** | **Clean reads (M)** | **Contigs**  **(> 300 bp)** | **N50 (bp)** | **Largest contig**  **(bp)** | **Gene catalogue** |
| --- | --- | --- | --- | --- | --- | --- |
| SCX1 | 97.84 | 96.25 | 624653 | 494 | 59907 | 3,407,679 |
| SCX2 | 111.00 | 108.86 | 279488 | 441 | 9835 |  |
| SCX3 | 85.54 | 83.85 | 124822 | 457 | 11782 |  |
| SCX4 | 92.11 | 90.32 | 212919 | 455 | 32398 |  |
| SCX5 | 95.19 | 93.44 | 235162 | 475 | 50326 |  |
| ZNZ1 | 137.01 | 134.96 | 925459 | 739 | 152355 |  |
| ZNZ2 | 96.68 | 94.96 | 410175 | 454 | 24885 |  |
| ZNZ3 | 101.94 | 100.22 | 669599 | 488 | 21887 |  |
| WY1 | 74.88 | 72.93 | 158375 | 413 | 23795 |  |
| WY2 | 92.09 | 89.85 | 189436 | 427 | 47092 |  |
| WY3 | 107.08 | 105.43 | 415746 | 454 | 15935 |  |

**Table S7** The alpha diversity indices (ACE, Chao1, Shannon and Simpson indices) of microbial community in sediment samples from water reservoirs.

| Sample names | ACE | Chao 1 | Shannon | Simpson |
| --- | --- | --- | --- | --- |
| SCX1 | 4282 | 4282 | 4.710515 | 0.028157 |
| SCX2 | 3681 | 3681 | 4.668319 | 0.027386 |
| SCX3 | 3487 | 3487 | 4.638067 | 0.026843 |
| SCX4 | 3576 | 3576 | 4.610889 | 0.030154 |
| SCX5 | 3664 | 3664 | 4.547978 | 0.032237 |
| ZNZ1 | 5161 | 5161 | 4.801166 | 0.040944 |
| ZNZ2 | 3401 | 3401 | 4.624423 | 0.02611 |
| ZNZ3 | 3529 | 3529 | 4.015637 | 0.05607 |
| WY1 | 3435 | 3435 | 4.78128 | 0.023495 |
| WY2 | 3455 | 3455 | 4.764469 | 0.023907 |
| WY3 | 3716 | 3716 | 4.868378 | 0.021707 |

**Table S8** 11 genera containing only nitrogen cycle functional genes and VBNC-related genes.

| No. | Genus |
| --- | --- |
| 1 | *Methylocystis* |
| 2 | *Gaiella* |
| 3 | *Methylobacter* |
| 4 | *Methanosarcina* |
| 5 | *Methanothrix* |
| 6 | *Methanoregula* |
| 7 | *Rhizobacter* |
| 8 | *Arthrobacter* |
| 9 | *Reyranella* |
| 10 | *Piscinibacter* |
| 11 | *Methylomonas* |

**
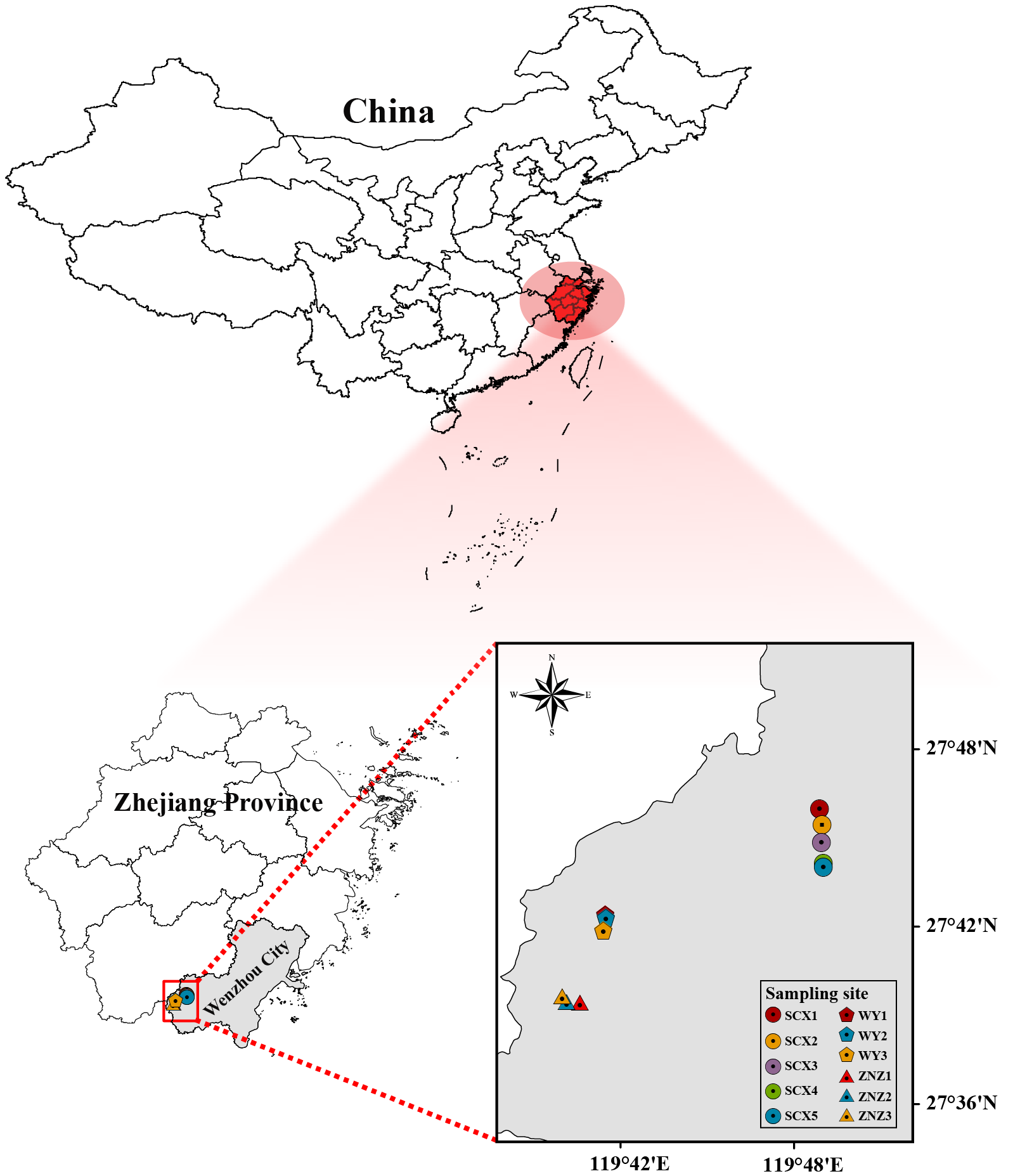
**

**Fig. S1.** Sampling locations in Reservoir Sediments of Wuyanling National Nature Reserve, Zhejiang Province.


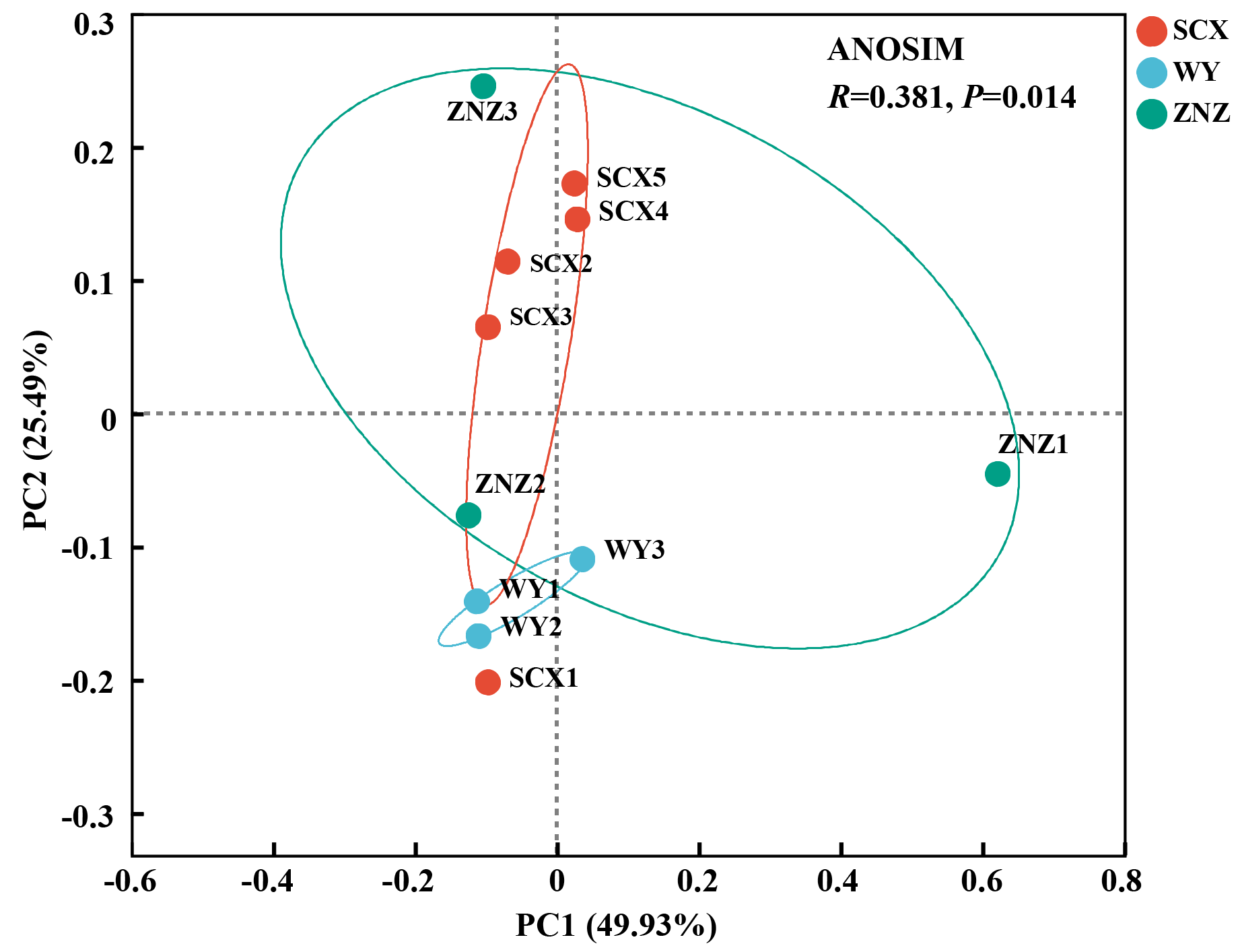


**Fig. S2.** Principal coordinate analysis (PCoA) of microbial community in sediment samples from water reservoirs using the unweighted UniFrac distance metric.


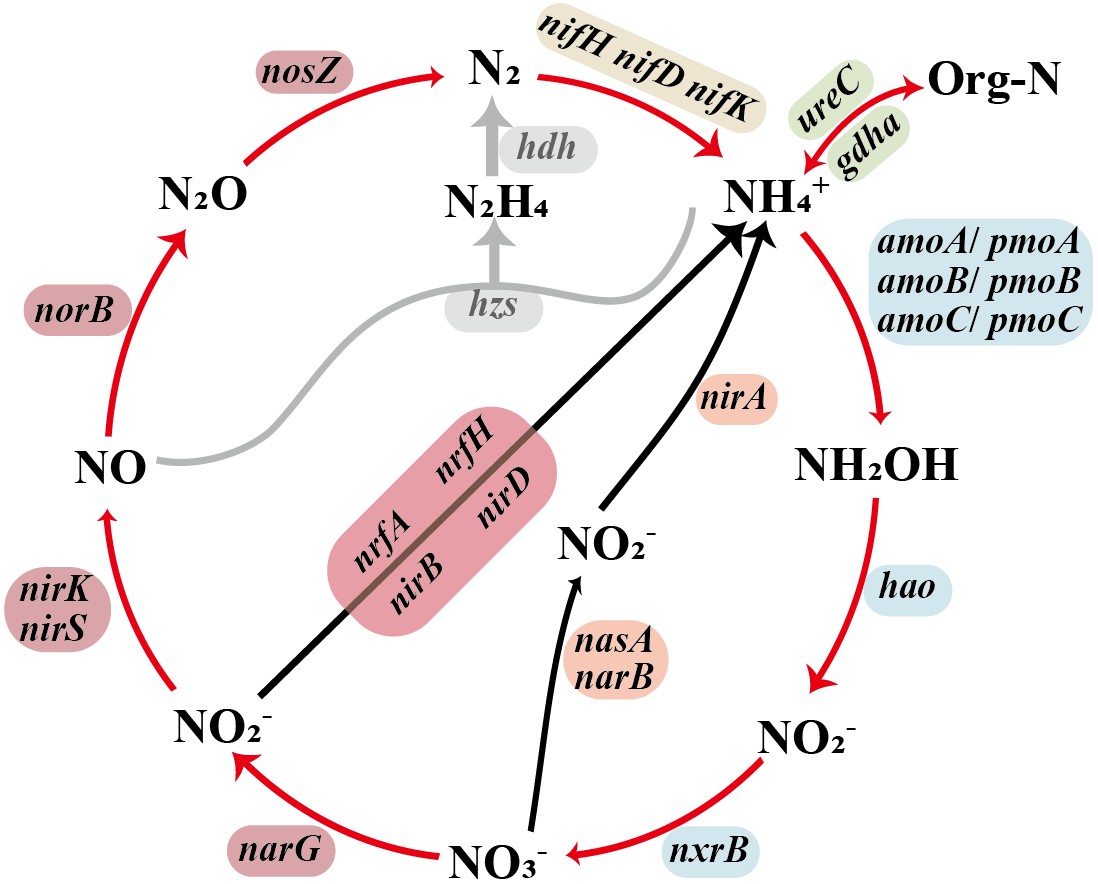


**Fig. S3.** Schematic diagram depicting the primary nitrogen transformation functions in reservoir sediments as revealed by KEGG annotation.

**
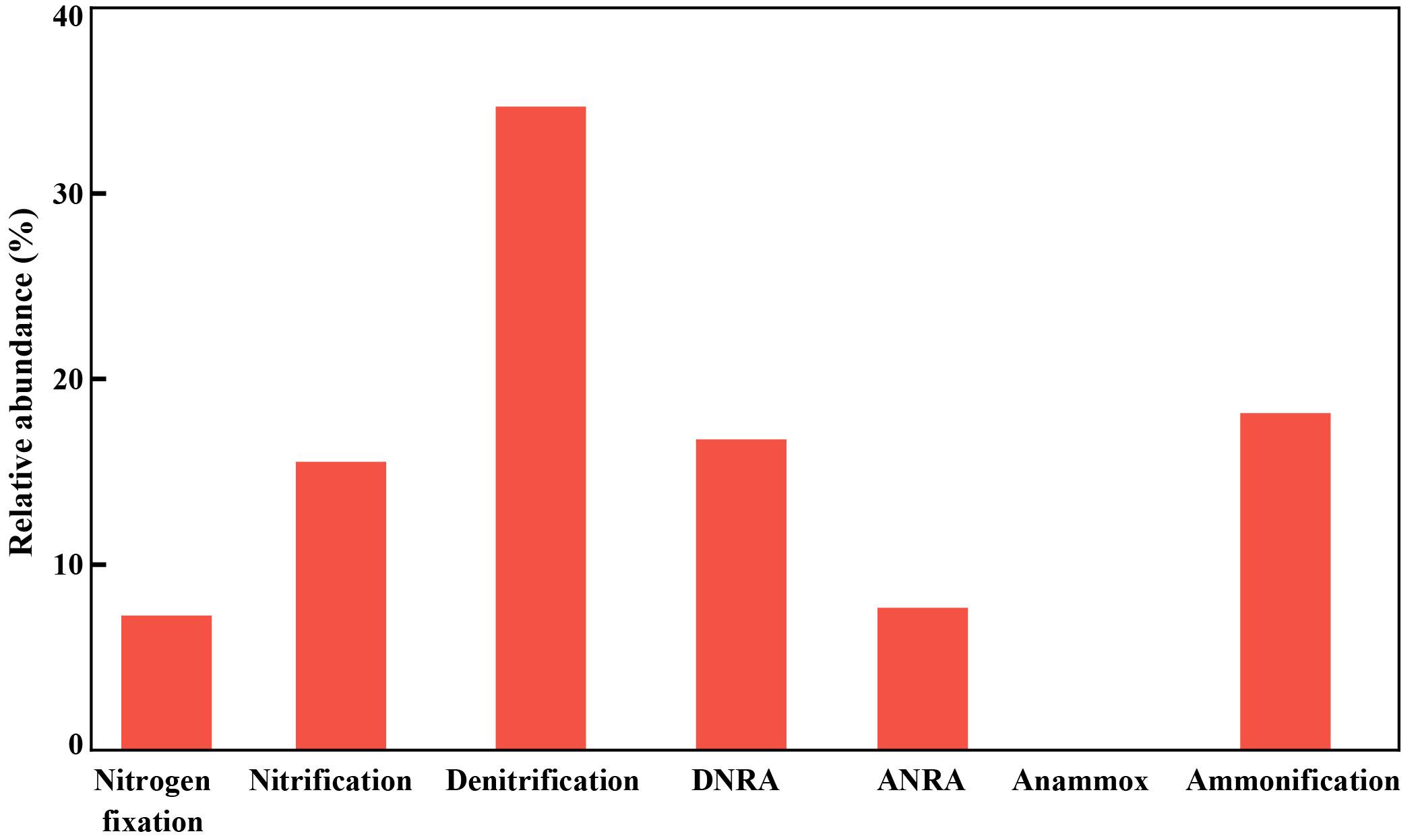
**

**Fig. S4.** Percentage of abundance for main nitrogen transformation functions in reservoir sediments. DNRA, dissimilatory nitrate reduction to ammonium; ANRA, assimilatory nitrate reduction to ammonium. Anammox, anaerobic ammonium oxidation.

**References**

Bai, K. H., Chen, X., Kan, Y. M., Jiang, N., Li, J. Q., Luo, L. X., 2022. Evaluation of optimal reference genes for the normalization by qPCR in viable but nonculturable state in *Xanthomonas campestris* pv. *campestris*. J. Phytopathol. 170, 399-407.

Cai, J. X., Zhou, M. Q., Zhang, Y. X., Ma, Y., Zhang, Y. B., Wang, Q. Y., 2023. Identification of determinants for entering into a viable but nonculturable state in *Vibrio alginolyticus* by Tn-seq. Appl. Microbiol. Biotechnol. 107, 1813-1827.

Chen, S., Li, X., Wang, Y. H., Zeng, J., Ye, C. S., Li, X. P., et al., 2018. Induction of *Escherichia coli* into a VBNC state through chlorination/chloramination and differences in characteristics of the bacterium between states. Water Res. 142, 279-288.

Dong, K., Pan, H. X., Yang, D., Rao, L., Zhao, L., Wang, Y. T., et al., 2020. Induction, detection, formation, and resuscitation of viable but non-culturable state microorganisms. Compr. Rev. Food Sci. Food Saf. 19, 149-183.

Dubey, V., Gupta, R., Pathania, R., 2021. Targeting Superoxide Dismutase Confers Enhanced Reactive Oxygen Species-Mediated Eradication of Polymyxin B-Induced *Acinetobacter baumannii* Persisters. Antimicrob. Agents Chemother. 65, 10.1128/aac.02180-20.

Gannon, V. P., D'Souza, S., Graham, T., King, R. K., Rahn, K., Read, S., 1997. Use of the flagellar H7 gene as a target in multiplex PCR assays and improved specificity in identification of enterohemorrhagic Escherichia coli strains. J. Clin. Microbiol. 35, 656-662.

Kusumoto, A., Asakura, H., Kawamoto, K., 2012. General stress sigma factor RpoS influences time required to enter the viable but non-culturable state in *Salmonella enterica*. Microbiol. Immunol. 56, 228-237.

Kwan, B. W., Chowdhury, N., Wood, T. K., 2015. Combatting bacterial infections by killing persister cells with mitomycin C. Environ. Microbiol. 17, 4406-4414.

Li, Y. X., Chen, X., Zhang, W. L., Fang, K. F., Tian, J. J., Li, F. Y., et al., 2024. The metabolic slowdown caused by the deletion of *pspA* accelerates protein aggregation during stationary phase facilitating antibiotic persistence. Antimicrob. Agents Chemother. 68, e00937-23.

Liao, H. B., Zhong, X. W., Xu, L., Ma, Q., Wang, Y. J., Cai, Y., et al., 2019. Quorum-sensing systems trigger catalase expression to reverse the *oxyR* deletion-mediated VBNC state in *Salmonella typhimurium*. Res. Microbiol. 170, 65-73.

Liao, X. Y., Hu, W. C., Liu, D. H., Ding, T., 2021. Stress resistance and pathogenicity of nonthermal-plasma-induced viable-but-nonculturable *Staphylococcus* aureus through energy suppression, oxidative stress defense, and immune-escape mechanisms. Appl. Environ. Microbiol. 87, e02380-20.

Loke, M. F., Ng, C. G., Vilashni, Y., Lim, J., Ho, B., 2016. Understanding the dimorphic lifestyles of human gastric pathogen *Helicobacter pylori* using the SWATH-based proteomics approach. Sci. Rep. 6, 26784.

Mohiuddin Sayed, G., Massahi, A., Orman Mehmet, A., 2022. *lon* Deletion Impairs Persister Cell Resuscitation in *Escherichia coli*. mBio 13, e02187-21.

Papadimitriou, K., Alegría, Á., Bron Peter, A., de Angelis, M., Gobbetti, M., Kleerebezem, M., et al., 2016. Stress physiology of lactic acid bacteria. Microbiol. Mol. Biol. Rev. 80, 837-890.

Pedersen, K., Christensen, S. K., Gerdes, K., 2002. Rapid induction and reversal of a bacteriostatic condition by controlled expression of toxins and antitoxins. Mol. Microbiol. 45, 501-510.

Shi, J., Zhou, X. R., Zhang, S. S., Sun, F. Q., Shen, C. F., Su, X. M., 2024. Unveiling the distribution characteristics of *rpf*-like genes and indigenous resuscitation promoting factor production in PCB-contaminated soils. J. Environ. Manage. 357, 120803.

Sulaiman, J. E., Hao, C. L., Lam, H., 2018. Specific enrichment and proteomics analysis of *Escherichia coli* persisters from rifampin pretreatment. J. Proteome Res. 17, 3984-3996.

Urbaniec, J., Xu, Y., Hu, Y., Hingley-Wilson, S., McFadden, J., 2022. Phenotypic heterogeneity in persisters: a novel ‘hunker’ theory of persistence. FEMS Microbiol. Rev. 46, fuab042.

Wang, H. W., Chung, C. H., Ma, T. Y., Wong, H. C., 2013. Roles of alkyl hydroperoxide reductase subunit C (AhpC) in viable but nonculturable *Vibrio parahaemolyticus*. Appl. Environ. Microbiol. 79, 3734-3743.

Wood, T. K., 2016. Combatting bacterial persister cells. Biotechnol. Bioeng. 113, 476-483.

Yu, C. G., Armengaud, J., Blaustein, R. A., Chen, K. Z., Ye, Z., Xu, F. J., et al., 2022. Antibiotic tolerance and degradation capacity of the organic pollutant-degrading bacterium *Rhodococcus biphenylivorans* TG9T. J. Hazard. Mater. 424, 127712.

Zhang, J. F., Wang, L., Shi, L., Chen, X., Chen, C. X., Hong, Z. C., et al., 2020. Survival strategy of *Cronobacter sakazakii* against ampicillin pressure: Induction of the viable but nonculturable state. Int. J. Food Microbiol. 334, 108819.

Zheng, M., Wang, X. D., Templeton Lori, J., Smulski Dana, R., LaRossa Robert, A., Storz, G., 2001. DNA microarray-mediated transcriptional profiling of the *Escherichia coli* response to hydrogen peroxide. J. Bacteriol. 183, 4562-4570.

Zhu, Y. G., Johnson, T. A., Su, J. Q., Qiao, M., Guo, G. X., Stedtfeld, R. D., et al., 2013. Diverse and abundant antibiotic resistance genes in Chinese swine farms. PNAS 110, 3435-3440.
